# Contact Allergy to Ingredients of Hair Cosmetics Associated with Occupational and Non‐Occupational Exposure—Trends from 1995 to 2020 in Central Europe, with or without Regulation

**DOI:** 10.1111/cod.70079

**Published:** 2025-12-30

**Authors:** Wolfgang Uter, Jakob Ferløv Baselius Schwensen, Brunhilde Blömeke, Olaf Gefeller, Swen M. John, Cara Bieck, Steffen Schubert

**Affiliations:** ^1^ Department of Medical Informatics Biometry and Epidemiology, University of Erlangen/Nürnberg Erlangen Germany; ^2^ National Allergy Research Centre, Department of Dermatology and Allergy Herlev and Gentofte Hospital, University of Copenhagen Hellerup Denmark; ^3^ Department of Environmental Toxicology Trier University Trier Germany; ^4^ Department of Dermatology Environmental Medicine and Health Theory, Osnabrück University Osnabrück Germany; ^5^ Institute for Interdisciplinary Dermatologic Prevention and Rehabilitation (iDerm) Osnabrück University Osnabrück Germany; ^6^ Division of Occupational Medicine, Department of Occupational Medicine Hazardous Substances and Health Sciences, Statutory Accident Insurance for the Health and Welfare Services Hamburg Germany; ^7^ Information Network of Departments of Dermatology Institute at the Georg‐August University, University Medical Center Göttingen Germany

**Keywords:** clinical epidemiology, contact allergy, hair cosmetics, hairdressers, regulation, RRID:SCR_001905, surveillance

## Abstract

**Background:**

Hair cosmetic products contain various chemicals which are partly potent contact sensitizers. These may cause occupational hand eczema in hairdressers, but also scalp and face dermatitis in consumers.

**Objective:**

To (i) highlight differences between the spectrum of occupational and non‐occupational exposure reflected in sensitization, and (ii) correlate trends in the last decades with regulatory interventions (or the lack thereof).

**Methods:**

Patch test and clinical data collected by the IVDK (https://www.ivdk.org) between 01/1995 and 12/2020 were subjected to a pooled re‐analysis. Age‐stratified sensitization prevalences in female hairdressers and consumers, respectively, were calculated for 2‐yearly intervals spanning the study period. Log‐binomial regression models were employed to estimate the association between contact allergy and (i) subgroup (hairdresser vs. consumer), (ii) a 3‐level age category, and/or (iii) time period, respectively.

**Results:**

Hair dyes were the most frequent sensitizers in hairdressers and consumers; however, there was a marked increase in (particularly the young) consumers. Pyrogallol had been banned in 1992 but still elicited positive patch test reactions even in the young. Hydroquinone, banned in 2013 in dyes, showed no decline in sensitization prevalence. Glyceryl thioglycolate has been withdrawn and later banned in Germany since around 1995, which caused contact allergy prevalence to drop to almost zero in the youngest age group, largely until today.

**Conclusion:**

As a limitation, putatively differing selection for patch testing render prevalences difficult to compare between hairdressers and consumers. The importance of hair dye allergy is evident, needing continued efforts to increase product safety from the manufacturing and regulatory side. The potential of (self‐)regulatory intervention is well illustrated by the decline of contact allergy to glyceryl thioglycolate.

## Graphical abstract

Among 2678 female hairdressers and 6244 female consumers with contact dermatitis suspected to be related to use of hair cosmetics, including dyes, patch tested in the IVDK between 1995 and 2020, diverging contact allergy trends were observed. In the case of toluene‐2,5‐diamine (PTD) shown in the figure, sensitization was at a stable, high level in hairdressers, whereas in female consumers up to age 32, a massive, significant increase was seen.

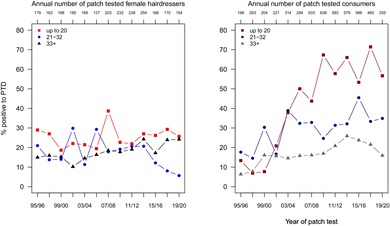

## 1 Introduction

Hair cosmetics have various functions, from everyday cleaning and conditioning to hair‐forming, bleaching, and (oxidative, i.e., permanent) hair dyeing, to name just the most common applications. These products lead to exposure of the scalp and glabrous skin in those whom they are applied to (consumers—including also hairdressers themselves) and of the hands, wrists and underarms in those who apply them (hairdressers and, to a varying, much lesser extent, consumers). Evidence regarding the quantitative differences in exposure between consumers and hairdressers has recently been summarized, indicating important excess exposure in hairdressers [1].

The multitude of hazardous hair cosmetic ingredients includes both “active” ingredients such as for dyeing, bleaching, and hair‐forming, and auxiliary ingredients like preservatives, scents (which may act as odour masks in some products which would otherwise emit an unpleasant odour), stabilizers, colourants, etc. Very frequently this leads to hand eczema in hairdressers [2], either of irritant or allergic origin, or of mixed aetiology. A recent systematic review has addressed contact allergy to important ingredients of hair cosmetics in hairdressers [3, 4, 5]. Consumers, much less exposed, may also acquire contact allergy to a product ingredient and consequently develop allergic contact dermatitis (ACD).

Risk management by the cosmetic industry, both in terms of self‐regulation (industry codes of practice) and legally binding regulation, as for example, provided by the Cosmetics Regulation in the European Union (EU; Cosmetics Regulation (EC) 1223/2009) [6] aims at mitigating such risks. Post‐marketing, epidemiological surveillance of cosmetic ingredients (or any other substance, for that matter) has repeatedly provided important input for reassessment of risk and rebalancing risk management. A time‐trend analysis is capable of demonstrating the effectiveness of regulation in terms of a decreasing contact allergy prevalence trend [7]. The present pooled re‐analysis of long‐term patch test and clinical data collected by the Information Network of Departments of Dermatology (https://ivdk.org/en) examines time trends of contact allergy to different hair cosmetic ingredients over a period of 26 years. During this period, some of these ingredients were subject to specific regulation, while most were not legally regulated. The objective of the long‐term analysis is to assess the impact, if any, of regulation, including possible reasons for observed trends or their lack. Secondly, differences between professional and consumer morbidity and their (possible) relation to differing exposure to the hair cosmetic ingredients considered will be examined.

## 2 Methods

The Information Network of Departments of Dermatology (IVDK; https://www.ivdk.org/en) is a tri‐national clinical surveillance network concerning contact allergy [8]. Scientifically, it is rooted in the German Contact Dermatitis Research group (DKG; https://dkg.ivdk.org/) with members also from Austria and the German‐speaking part of Switzerland. Like many contact dermatitis societies, the DKG maintains a “hairdresser” or “hair cosmetic” patch test series which includes the main allergens (haptens) considered here. This series has undergone several changes since the beginning of the study period in 1995 and currently corresponds largely to a European recommendation, dating from 2015 [9]. Mostly, the baseline series has been tested in addition, including several preservatives and fragrance screening allergens of interest, also with some variation in scope over time.

The participating IVDK departments are mostly located in Germany (*n* = 62), followed by Switzerland (*n* = 6) and Austria (*n* = 3). All centres are members of the DKG and adhere to European Society of Contact Dermatitis (ESCD) patch test guidelines [10], with slight refinement [11, 12]. The departments collect patch test data along with clinical data and important items of the patient's history electronically, and transmit these data twice yearly to the IVDK central office in Göttingen for quality checks [13] and eventual pooling. The present period covers data obtained from January 1995 to December 2020. Following the methodology of the previous four single studies [14, 15, 16, 17], patients were stratified into one of the following two subgroups:

1. Hairdressers included all female patients either currently working as hairdressers and diagnosed with occupational dermatitis, or previously suffering from occupational dermatitis when working as a hairdresser.
2. Consumers included all female patients in whom hair cosmetics were suspected as the cause of contact dermatitis (up to three causes could be recorded per patient), and who had never worked as hairdressers, according to the case documentation. “Consumers” include clients of hairdressing salons as well as self or home users, that is, patients who applied hair cosmetic products such as hair dyes themselves or had these applied by non‐professional helpers.

As in the previous analyses, only female patients were included to avoid confounding of results by sex and the necessity of additional adjustment for sex, respectively. In the present data, *n* = 226 (7.8%) of hairdressers fulfilling above case definition, and *n* = 470 (7%) of “consumers” were male. The two large female subgroups were compared concerning demographic and clinical characteristics. This mainly concerned the prevalence of sensitization to allergens included in the “hairdresser series” of the DKG (versions since 1995 see Table S1). The patch test preparation ammonium thioglycolate 1% pet. used until 2002 was disregarded owing to doubts concerning diagnostic usefulness. Furthermore, sensitization to preservatives potentially found in hair cosmetics tested as part of the baseline test series [18] was analysed. To address time trends across 2‐year intervals, patch test positivity was stratified for age groups ≤ 20, 21–32, and > 32, both for hairdressers and consumers. Patch test preparations were purchased from Almirall Hermal, Reinbek, Germany (until 2013), SmartPractice Europe, Greven, Germany (from 2014 on), and partly from Chemotechnique, Vellinge, Sweden. Patch test exposure time was 2 days in most departments (75%) and 1 day in the remainder (25%) during the study period. Readings on the third day (D3) after the start of application of the patches or, if these were not performed, on the fourth day (D4) were aggregated to the outcome ‘positive’ (+, ++ and +++ reactions; i.e., erythema, infiltration, papules and/or (coalescing) vesicles) versus ‘non‐positive’ (doubtful, irritant and negative reactions). Overall, the substitution of a D3 with a D4 reading is noted in 1.35% of the patients—thus, rare. Prevalence estimates were age‐stratified, considering the youngest age stratum to approximate incidence.

For the quantitative summary of possible effects of interest which are all based on the fully stratified descriptive tabulated results, multiple log‐binomial regression modelling was employed for each of the allergens presented here. A positive (vs. non‐positive) patch test reaction was taken as a dichotomous outcome. The explanatory factors included (i) being a female consumer (vs. a female hairdresser), (ii) the age group, using the oldest (> 32 years of age) as reference, and (iii) the time trend, modelled linearly across the 2‐year intervals with the initial period as reference. Moreover, log‐binomial regression analysis was stratified for exposure group to examine a possibly diverging pattern of association between specific sensitization and time period and age group, respectively. Effects were quantified as prevalence ratio (PR) and accompanied by 95% confidence intervals (CIs) estimated using the profile likelihood method. The PR can be interpreted as a relative risk, that is, a factor in‐ or decreasing the prevalence seen in a particular subgroup relative to the corresponding reference prevalence. In addition, solely for the estimation of the in‐ or decrease of age across the time periods, a simple linear regression analysis stratified by subgroup was performed.

For data management at the Göttingen data centre, SAS software (version 9.4; SAS Institute, Cary, NC) was used. For data analysis following a guideline for the descriptive analysis of contact allergy data [19] the R statistical software package (version 4.4.1, https://www.r‐project.org/, RRID:SCR_001905) was utilized.

## 3 Results

### 3.1 Demographics

Between January, 1995, and December, 2020, 276 113 consultations of 265 823 patients have been documented by the departments of the IVDK network. Thereof, 175 690 consultations were by 169 218 female patients, corresponding to 63.7% of all patients. Across the study period, the annual share of female patients fluctuated slightly between 66.3% and 63.9% until 1998, remained on a lower level between 61.1% and 62.6% 1999–2008, and finally on a higher level between 63.6% and 65.4% in the remaining period. The distribution of female patients across the 2‐year periods in the two subgroups of hairdressers (*N* = 2678) and consumers (*N* = 6244), respectively, is shown in Table S2, including a breakdown per country and accompanied by the age medians in hairdressers and consumers, respectively, in these 2‐year periods. The overall median amounted to 25 years in hairdressers and 49 years in consumers (*p* < 0.00001, Wilcoxon test). Among all female patients patch tested, the share of hairdressers decreased just very slightly, if significantly (*p* = 0.004, Cochrane‐Armitage trend test), whereas the share of consumers increased overall (*p* < 0.00001, Cochrane‐Armitage trend test), albeit not in terms of a steady trend and with some decrease in the last years of the study period (Figure S1). The pattern of dermatitis was characteristically different between hairdressers (predominantly hand dermatitis) and consumers (predominantly head/face/neck dermatitis); for further details, see Table 1. Using linear regression analysis on hairdressers and consumers each, a significant (*p* < 0.00001, regression *t*‐test) overall increasing linear age trend by a—virtually identical—average of 0.43 years of age per 2‐year period was identified.

**TABLE 1:** Description of demographic and clinical characteristics of female hairdressers and consumers patch tested in the IVDK between 01/1995 and 12/2020 according to the MOAHLFA index with Data S1.

	Hairdressers	Consumers
	(*n* = 2678)	(*n* = 6244)
Patch test: 1995–2008 (*n*)	1313	2523
2009–2020 (*n*)	1365	3721
Age in years (Q1; median; Q3)	20; 25; 40	35; 49; 60
Proportion aged ≥ 40 years (%)	25	67.8
Current occupational skin disease (%)	86	3
Past or current atopic dermatitis (%)	36.6	19.7
Main involved site (%)		
Hand	82.5	7.6
Leg	0.3	1.2
Head, face, or neck	7.3	71.4
Other	9.9	19.8
Tested with (%):		
Baseline series	91.9	92.6
Hairdresser series	93.4	84.3

### 3.2 Oxidative Hair Dye Primary and Secondary Intermediates

#### 3.2.1 Toluene‐2,5‐Diamine (PTD)

**FIGURE 1:**
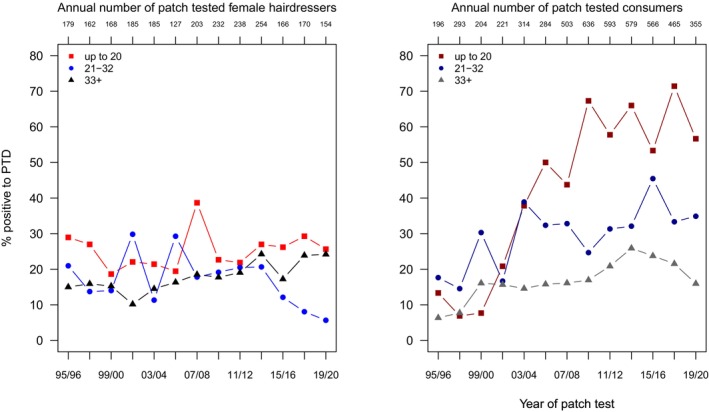
Time trend of positive patch test reactions (crude, age‐stratified prevalences) to toluene‐2,5‐diamine (PTD) 1% pet. in female hairdressers (left) and female consumers (right) consulting the departments of the IVDK between 1995 and 2020.

From the descriptive results in Table S3A and Figure 1, a higher frequency of sensitization in consumers than in hairdressers since 2003/04 is evident. In the fully adjusted regression model, that is, with period and age group as additional covariates, being a consumer entailed a risk increased by 46% with a PR (95% CI) of 1.46 (1.32–1.62) compared to being a hairdresser (Table 2). Looking next at the subgroup of hairdressers, no increase of risk was found during the study period (PR 1.01 (0.98–1.03)). Compared to the oldest subgroup, younger hairdressers had a significantly increased risk of being sensitized (PR 1.41 (1.16–1.71)), while the middle age group was similar to the oldest (reference) group (Table 3). In consumers, conversely, a significant average increase of sensitization prevalence over the study period of 8% every 2 years was found. Of note, the middle age group showed an increased risk of being sensitized to PTD (PR 1.7 (1.49–1.92)), which was even higher in the youngest group (PR 2.69 (2.39–3.01)) compared to the oldest.

**TABLE 2:** Results of a multifactorial log‐binomial regression analysis with positive (vs. non‐positive) reaction to the allergen (in rows) as outcome, and exposure subgroup (with hairdressers as reference), period (per 2 years, with 1995/96 as reference), and age group (with age > 32 years as reference) as explanatory factors.

Allergen	Consumers	Period	Age < 21	Age 21–32	Age > 32
PTD	1.46 (1.32–1.62)	1.05 (1.04–1.07)	2.33 (2.09–2.58)	1.50 (1.34–1.67)	1.00 (ref.)
PPD	1.57 (1.41–1.76)	1.06 (1.05–1.08)	2.03 (1.79–2.28)	1.50 (1.34–1.68)	1.00 (ref.)
pAP	4.56 (3.76–5.58)	1.09 (1.07–1.11)	6.40 (5.48–7.45)	3.03 (2.54–3.61)	1.00 (ref.)
mAP	5.78 (4.46–7.62)	1.10 (1.08–1.13)	6.87 (5.64–8.34)	3.24 (2.60–4.03)	1.00 (ref.)
Hyqu.	12.9 (8.04–22.4)	1.11 (1.06–1.16)	31.7 (21.7–47.9)	8.02 (5.10–12.8)	1.00 (ref.)
Pyro.	2.15 (1.65–2.83)	0.94 (0.91–0.97)	4.84 (3.61–6.48)	3.02 (2.24–4.07)	1.00 (ref.)
APS	0.16 (0.13–0.20)	0.96 (0.95–0.98)	2.49 (2.07–3.01)	1.41 (1.15–1.73)	1.00 (ref.)
ATG	0.42 (0.23–0.79)	1.10 (0.96–1.26)	0.88 (0.34–2.02)	1.06 (0.51–2.12)	1.00 (ref.)
GMTG	0.20 (0.15–0.26)	0.84 (0.81–0.87)	0.88 (0.65–1.19)	0.73 (0.53–0.99)	1.00 (ref.)
MCI/MI	0.32 (0.25–0.41)	1.06 (1.03–1.10)	0.54 (0.36–0.77)	0.50 (0.35–0.70)	1.00 (ref.)
MDBGN^a^	0.41 (0.32–0.54)	0.93 (0.90–0.96)	0.40 (0.26–0.59)	0.48 (0.33–0.67)	1.00 (ref.)

*Note:* Association quantified by prevalence ratios (PRs) with accompanying 95% confidence intervals (CIs).

Abbreviations: APS, ammonium persulfate; ATG, ammonium thioglycolate; GMTG, glyceryl thioglycolate; Hyqu., hydroquinone; mAP, *m*‐aminophenol; MCI/MI, methychloroisothiazolinone/methylisothiazolinone 3:1; MDBGN, methyldibromo glutaronitrile; pAP, *p*‐aminophenol; PPD, *p*‐phenylenediamine; PTD, toluene‐2,5‐diamine; Pyro., pyrogallol.

^a^ Additionally adjusted for test concentration which varied over time.

**TABLE 3:** Results of a stratified multifactorial log‐binomial regression analysis, subdivided into the subgroups of hairdressers and consumers, respectively, with positive (vs. non‐positive) reaction to the allergen (in rows) as outcome, and period (per 2 years, with 1995/96 as reference) as well as age group (with age > 32 years as reference) as explanatory factors.

Allergen	Hairdressers			Consumers		
	Period	Age < 21^b^	Age 21–32^b^	Period	Age < 21^b^	Age 21–32^b^
PTD	1.01 (0.98–1.03)	1.41 (1.16–1.71)	0.95 (0.77–1.16)	1.08 (1.06–1.09)	2.69 (2.39–3.01)	1.70 (1.49–1.92)
PPD	1.02 (1.00–1.05)	1.33 (1.06–1.68)	1.15 (0.92–1.44)	1.08 (1.06–1.10)	2.33 (2.04–2.64)	1.57 (1.38–1.79)
pAP	1.02 (0.97–1.08)	1.84 (1.17–2.95)	1.21 (0.75–1.98)	1.10 (1.08–1.13)	7.13 (6.10–8.33)	3.22 (2.67–3.88)
mAP	1.06 (0.99–1.14)	1.16 (0.63–2.17)	0.97 (0.53–1.80)	1.11 (1.08–1.14)	7.99 (6.53–9.74)	3.50 (2.76–4.40)
Hyqu.	1.11 (0.97–1.29)	4.30 (1.04–28.9)	3.33 (0.81–22.3)	1.11 (1.06–1.16)	34.7 (23.5–53.1)	7.82 (4.84–12.8)
Pyro.	0.89 (0.83–0.94)	1.87 (1.03–3.55)	2.17 (1.22–4.05)	0.97 (0.93–1.01)	6.56 (4.78–8.96)	2.86 (1.98–4.06)
APS	0.97 (0.95–0.99)	2.61 (2.12–3.24)	1.40 (1.11–1.78)	0.93 (0.89–0.98)	1.70 (0.98–2.76)	1.65 (1.04–2.51)
ATG	1.08 (0.89–1.31)	0.37 (0.08–1.18)	0.75 (0.30–1.79)	1.11 (0.92–1.35)	2.45 (0.71–6.57)	1.41 (0.41–3.79)
GMTG	0.79 (0.75–0.82)	0.85 (0.62–1.16)	0.66 (0.46–0.93)	0.97 (0.90–1.04)	0.79 (0.28–1.78)	1.24 (0.64–2.22)
MCI/MI	1.07 (1.02–1.13)	0.48 (0.30–0.73)	0.42 (0.27–0.63)	1.05 (1.00–1.11)	0.70 (0.31–1.33)	0.71 (0.39–1.19)
MDBGN^a^	0.85 (0.81–0.90)	0.24 (0.13–0.41)	0.49 (0.32–0.74)	1.01 (0.96–1.06)	0.89 (0.47–1.53)	0.39 (0.19–0.73)

*Note:* Association quantified by prevalence ratios (PRs) with accompanying 95% confidence intervals (CIs).

Abbreviations: APS, ammonium persulfate; ATG, ammonium thioglycolate; GMTG, glyceryl thioglycolate; Hyqu., hydroquinone; mAP, *m*‐aminophenol; MCI/MI, methychloroisothiazolinone/methylisothiazolinone 3:1; MDBGN, methyldibromo glutaronitrile; pAP, *p*‐aminophenol; PPD, *p*‐phenylenediamine; PTD, toluene‐2,5‐diamine; Pyro., pyrogallol.

^a^ Additionally adjusted for test concentration which varied over time.

^b^ Age reference: Age > 32 years.

#### 3.2.2 *p*‐Phenylenediamine (PPD)

The trend of contact allergy to PPD is illustrated in Figure S2 and Table S3B. The full regression model including both subgroups as factors shows all effects to be significant (Table 2). Stratifying analyses for group, only a slight, non‐significant increase over time can be seen in hairdressers (2% increase per 2 years, PR 1.02 (1–1.05)). While the youngest hairdressers had a moderately increased risk (PR 1.33 (1.06–1.68)), risk did not differ significantly between the oldest age group (reference) and the middle age group (Table 3). In consumers, a different picture evolves: the effect for period is significant and also more marked (PR 1.08 (1.06–1.1)). Both the young (PR 2.33 (2.04–2.64)) and the middle (PR 1.57 (1.38–1.79)) age group show a considerably increased risk compared to the oldest subgroup of consumers.

#### 3.2.3 *p*‐Aminophenol

**FIGURE 2:**
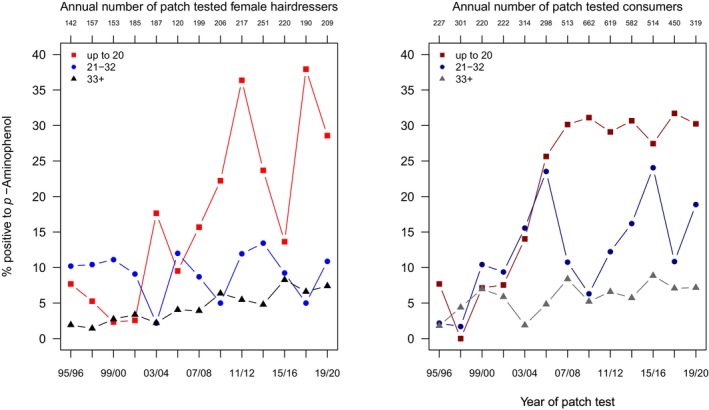
Time trend of positive patch test reactions (crude, age‐stratified prevalences) to *p*‐aminophenol 1% pet. in female hairdressers (left) and female consumers (right) consulting the departments of the IVDK between 1995 and 2020.

The descriptive results indicate that this primary intermediate, *p*‐aminophenol, is a somewhat less common sensitizer compared to PTD and PPD, especially in hairdressers (Table S3C, Figure 2). On a general level, the increased frequency of sensitization in consumers is striking (PR 4.56 (3.76–5.58)), see Table 2. Across both subgroups, younger patients exhibit a marked increase in risk to be found sensitized, namely a PR of 3.03 (2.54–3.61) in the middle age group, and of 6.4 (5.48–7.45) in the youngest age group. Moreover, a just slightly more pronounced increasing overall time trend (PR 1.09 (1.07–1.11)) was identified. In the subgroup of hairdressers, the only significant effect was a moderate excess of risk in the youngest age group (PR 1.84 (1.17–2.95)). Accordingly, much of the dynamics and contrasts seen overall are contributed by the subgroup of consumers: a 10% increase in risk per 2 years as well as a marked inverse age gradient, with PR 3.22 (2.67–3.88) for the middle and PR 7.13 (6.1–8.33) for the youngest age group.

#### 3.2.4 *m*‐Aminophenol

**FIGURE 3:**
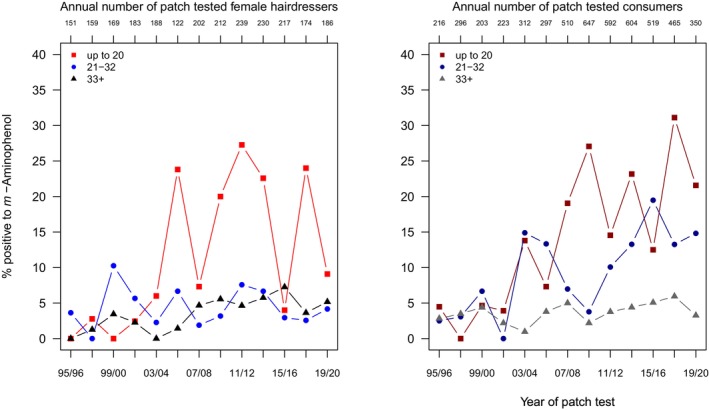
Time trend of positive patch test reactions (crude, age‐stratified prevalences) to *m*‐aminophenol 1% pet in female hairdressers (left) and female consumers (right) consulting the departments of the IVDK between 1995 and 2020.

Regarding this secondary intermediate, the difference in the level of sensitization between the two subgroups noted with *p*‐aminophenol is even more pronounced, as revealed in Table S4A, illustrated in Figure 3, and quantified by a PR of 5.78 (4.46–7.62) for sensitization risk in consumers as compared to hairdressers. Further, a considerable overall time trend is seen (PR 1.1 (1.08–1.13)) per 2‐year interval, and a marked age gradient, with a PR of 3.24 (2.6–4.03) for the middle, and of 6.87 (5.64–8.34) for the youngest age group. Specifically analyzing hairdressers, neither time trend nor age gradient is significant (Table 3). Consumers, conversely, apparently drive the effects observed overall, with a period effect of 11% increase per two years, and a marked age gradient with a PR of 3.5 (2.76–4.4) in the middle and 7.99 (6.53–9.74) in the youngest age group.

#### 3.2.5 Hydroquinone

**FIGURE 4:**
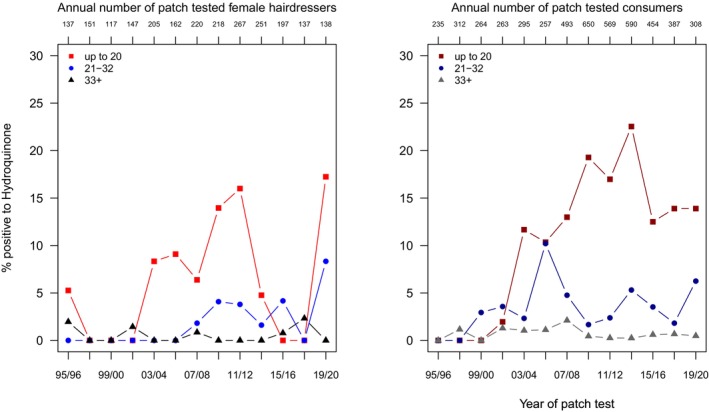
Time trend of positive patch test reactions (crude, age‐stratified prevalences) to hydroquinone 1% pet. in female hairdressers (left) and female consumers (right) consulting the departments of the IVDK between 1995 and 2020.

Hydroquinone was removed as a test allergen in the hair cosmetics series mid 2015 (Table S1) but maintained in the DKG adhesives and resin series and was thus available for patch testing (Table S4B, Figure 4). The overall regression analysis (Table 2) results indicate a marked difference between groups, with consumers being 12.9 (8.04–22.4) times more likely to be diagnosed sensitized. Moreover, the time trend shows a 10% increase in risk per 2 years, and a considerable age gradient: PR 8.02 (5.1–12.8) for the middle and PR 31.7 (21.7–47.9) for the youngest age group overall. Focusing on hairdressers, no significant effects are found except for a significant increase in risk in the youngest age group (PR 4.3 (1.04–28.9)) compared to the oldest age group. In consumers, however, driving again the overall effects observed, the period effect (PR 1.11 (1.06–1.16)) and the age effect (PR of 7.82 (4.84–12.8) in the middle and of 34.7 (23.5–53.1) in the youngest age group) were quite strong (Table 3).

#### 3.2.6 Pyrogallol

The “persistence” of pyrogallol sensitization is shown in Table S4C and Figure S3. The overall regression analysis reveals a picture similar to the previous secondary intermediates (if on a less pronounced level), except for a significant overall decline of sensitization frequency (PR 0.94 (0.91–0.97), i.e., a decrease by 6% each 2 years), see Table 2. Being a consumer was again associated with an increased risk (PR 2.15 (1.65–2.83)), as was younger age, with a PR of 3.02 (2.24–4.07) in the middle and 4.84 (3.61–6.48) in the youngest age group. In the subgroup of hairdressers, the period effect is 11% decrease per 2 years (Table 3). The age effect in this subgroup indicates an increased risk (compared to the oldest hairdressers) in the middle age group (PR 2.17 (1.22–4.05)) and, somewhat less so, in the youngest group (PR 1.87 (1.03–3.55)). In consumers, the time trend is much weaker with a non‐significant decrease of 3%, whereas the age trend is similar to that seen in *m*‐aminophenol, with a PR 2.86 (1.98–4.06) for the middle, and PR 6.56 (4.78–8.96) for the youngest age group.

#### 3.2.7 Overall Burden of Sensitization to Oxidative Hair Dye Ingredients

In order to address both a global trend of “hair dye component” contact allergy and possible differences between hairdressers and consumers, the number of contact allergies, if any, to the five above‐mentioned dye components (excluding pyrogallol) was individually calculated. The percentages of different numbers of positive reactions significantly differed between hairdressers and consumers (*p* = 0.0005, Fisher's exact test), with 73.7% hairdressers (72.1% consumers) without any positive reaction, 12.5% (8.6%) one, 9.7% (8.6%) two, 2.4% (3.9%) three, 1.5% (4.5%) four, and 0.2% (2.3%) five positive reactions, that is, to each agent considered here.

To corroborate this impression, a log‐binomial regression analysis was performed as before, with “any dye reaction” (among the five dye components considered) as outcome. The overall analysis revealed (i) a significant increase of 4% per 2‐year interval (PR: 1.04 (1.03–1.06)), (ii) a higher risk in consumers (PR 1.28 (1.16–1.41)), and (iii) an age gradient as observed in most single substances (see above) with a PR 1.35 (1.21–1.51) in the middle and 1.79 (1.60–2.00) in the youngest age group. Among hairdressers, no significant time trend was observed (PR 1.01 (0.99–1.03)), and only the youngest age group displayed a slightly and significantly increased risk (PR 1.31 (1.09–1.58)). Consumers, in return, showed a more pronounced time trend (PR 1.06 (1.05–1.08), i.e., a 6% increase per 2 years). The age gradient, too, was more pronounced than in hairdressers, with PR 1.47 (1.29–1.67) for middle and 2.05 (1.8–2.32) for the youngest consumers, compared to the oldest group.

Another perspective on the burden of oxidative hair dye‐related contact allergies is provided by examining cross‐reactivity. In Table 4, coupled reactivity between PPD and the five other compounds is illustrated by calculating the %ages of positive (and negative) patch test reactions to the other compounds in subgroups with negative, weak (+) positive, and extreme (+++) positive patch test reactions to PPD, respectively (doubtful and ++ positive reactions have been omitted for clarity). A general trend of a higher share of co‐reactivity with +++ reactions to PPD is evident.

**TABLE 4:** Pattern of cross‐reactivity between *p*‐phenylenediamine (PPD) in columns, contrasting negative (neg.), weak positive (+; erythema and infiltration, possibly papules), and extreme positive (+++; coalescing vesicles additionally to “+”) patch test reactions, respectively, and five primary and secondary intermediates, contrasting negative and positive (+, ++, or +++) reactions, in rows.

		PPD: neg.	PPD: +	PPD: +++
PTD	neg.	4010 (88.1%)	166 (34.9%)	18 (3.6%)
	+, ++ or +++	273 (6%)	253 (53.3%)	484 (95.8%)
p‐AP	neg.	4495 (98.5%)	412 (85.7%)	138 (27.4%)
	+, ++ or +++	38 (0.8%)	40 (8.3%)	347 (68.8%)
m‐AP	neg.	4528 (99.3%)	456 (95%)	230 (45.5%)
	+, ++, or +++	19 (0.4%)	16 (3.3%)	252 (49.8%)
HQ	neg.	4271 (98.5%)	447 (96.5%)	329 (74.8%)
	+, ++, or +++	19 (0.4%)	5 (1.1%)	91 (20.7%)
Pyro.	neg.	4152 (95.4%)	426 (91%)	390 (83%)
	+, ++, or +++	91 (2.1%)	22 (4.7%)	61 (13%)

*Note:* Percentages are column percentages, relating to all patients reacting negative, +, and +++, respectively, to PPD, also being tested with the respective other substance, excluding irritant or doubtful reactions.

Abbreviations: HQ, hydroquinone; m‐AP, *m*‐aminophenol; p‐AP, *p*‐aminophenol; PTD, toluene‐2,5‐diamine (*p*‐toluenediamine); Pyro., pyrogallol.

### 3.3 Bleaching Agent

**FIGURE 5:**
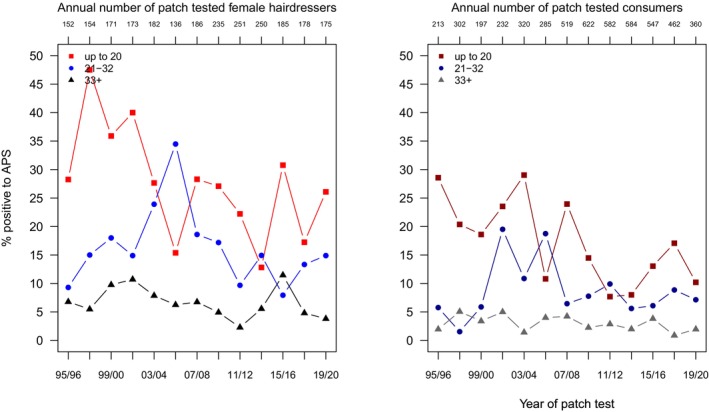
Time trend of positive patch test reactions (crude, age‐stratified prevalences) to ammonium persulfate (APS) 2.5% pet. in female hairdressers (left) and female consumers (right) consulting the departments of the IVDK between 1995 and 2020.

Ammonium persulfate (APS) has been tested in the DKG hairdresser series throughout the study period. The descriptive patch test results shown in Table S5 indicate (i) a much higher prevalence in hairdressers compared to consumers, (ii) a higher sensitization prevalence in the younger subgroups, and (iii) a decline over time which is also evident from Figure 5. Quantifying these observations by log‐binomial regression analysis, the probability to diagnose sensitization to APS decreased slightly (PR 0.96 (0.95–0.98)) but significantly per each 2‐year period (Table 2). Compared to hairdressers, consumers have a much lesser risk (PR 0.16 (0.13–0.20)) – or, conversely, hairdressers have a much higher risk to be sensitized than consumers (PR: 6.25). In the subgroup of hairdressers, a very modest but significant decline of sensitization to APS was seen, with a 3% decrease per 2 years. The age gradient indicates a relatively moderate increase in risk in the middle age group (PR 1.40 (1.11–1.78)) and a somewhat more pronounced increase in the youngest (PR 2.61 (2.12–3.24)); Table 3. In consumers, a significant average biannual decrease by 7% was found, and a lesser age gradient than in hairdressers: PR 1.65 (1.04–2.51) in the middle and PR 1.70 (0.98–2.76) in the youngest subgroup.

### 3.4 Waving/Relaxing Agents

#### 3.4.1 Ammonium Thioglycolate

As can be seen from Table S6A and Figure S4, sensitization is rare, both in hairdressers and consumers. The overall regression analysis revealed, as only significant result, hairdressing as a risk factor (PR 2.38) or, conversely, a significantly lesser risk in consumers (PR 0.42 (0.23–0.79)), see Table 2. Neither within the subgroup of hairdressers nor in consumers was any of the risk factors examined significant.

#### 3.4.2 Glyceryl Thioglycolate (GMTG)

**FIGURE 6:**
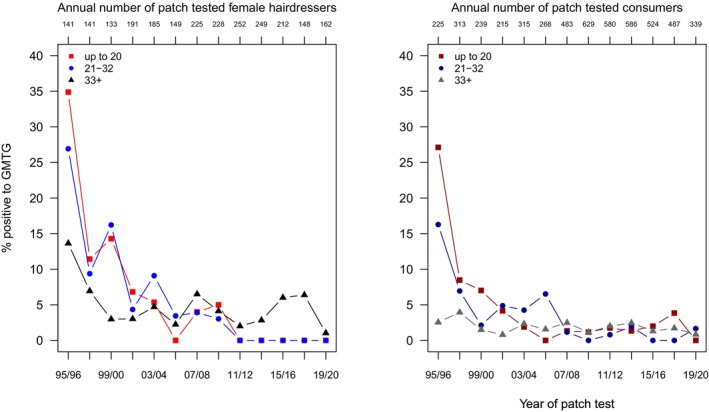
Time trend of positive patch test reactions (crude, age‐stratified prevalences) to glyceryl thioglycolate (GMTG) 1% pet. in female hairdressers (left) and female consumers (right) consulting the departments of the IVDK between 1995 and 2020; regarding hairdressers, see also [17, 19].

The overall regression analysis revealed a significantly lesser risk in consumers compared to hairdressers (PR 0.20 (0.15–0.26)) and, accordingly, a much higher risk in hairdressers compared to consumers (PR 5). Furthermore, an overall decrease by 16% by each 2‐year period is seen, well in line with the descriptive results in Table S6B/Figure 6. However, the linear representation of the time trend by one averaging factor in the standard analysis does not describe the shape of decline well, which is evident from a comparison with Figure 6. Furthermore, the overall observed negative age trend, that is, a lesser risk in young (PR 0.88 (0.65–1.19)) and middle aged (PR 0.73 (0.53–0.99)) patients does not take into account the fact that initially, the youngest hairdressers were most affected, while since about 2005 these are almost no longer affected, while middle aged and especially older hairdressers, owing to a cohort effect, are still diagnosed with “historical” GMTG sensitization, thus reversing the age gradient (Figure 6).

Focusing on the subgroup of hairdressers with the youngest age of up to 20 years, positive patch test results were still observed after 2004, that is, when exposure even in the 20‐year‐old should have ceased in Germany, following the ban of GMTG in 1995. As generally the results are pooled over the three countries, a more detailed view is warranted here, revealing 1 case from Austria, 2 cases in Switzerland, and also 5 in Germany.

### 3.5 Auxiliary Substances

According to previous analyses, biocides (preservatives) are the allergens of most concern among the “ubiquitous” (hair) cosmetic product ingredients.

#### 3.5.1 Methylchloroisothiazolinone/Methylisothiazolinone (MCI/MI)

Methylisothiazolinone (MI) alone has been included in the German baseline series only since January 2014, hence the mixture with methylchloroisothiazolinone (MCI), that is, MCI/MI, was considered for this long‐term analysis (Table S7A, Figure S5). The overall regression analysis revealed a much lesser risk of consumers to be sensitized (PR 0.32 (0.25–0.41)) or of a 3.12‐fold increase of risk in hairdressers, respectively (Table 2). The time trend—an average increase by 6% per 2 years—cannot sensibly be interpreted, in view of the intercurrent MI contact allergy epidemic around 2013, which is reflected, to some extent, also in the MCI/MI results (see discussion). Compared to the oldest age group (defined as age 33 years or above here), the middle age group (PR 0.5 (0.35–0.7)) and the youngest age group (PR 0.54 (0.36–0.77)) had a significantly lower risk to be sensitized. Disregarding the time trend factor (see above), results in hairdressers were somewhat more pronounced, with the middle age group (PR 0.42 (0.27–0.63)) and the youngest age group (PR 0.48 (0.30–0.73)) having an about halved risk compared to the oldest subgroup (Table 3). In consumers, by contrast, the PR 0.71 (0.39–1.19) in the middle and PR 0.70 (0.31–1.33) in the youngest age group did not reach significance.

#### 3.5.2 Methyldibromo Glutaronitrile (MDBGN)

The patch test concentration for MDBGN tested in the baseline series has fluctuated several times between 0.2% pet. and 0.3% pet. (see Table S7B and Figure S6). An increase of the patch test concentration from 0.2% to 0.3% pet. had, in the study period 2010–2017, about doubled the yield of positive patch test reactions [20]. Thus, it was considered necessary to adjust the present analysis additionally for patch test concentration as a confounder to be able to examine the time trend validly. A full regression model thus further adjusted identified a 2.44‐fold increased risk in hairdressers, the inverse of the estimate for consumers shown in Table 2. In terms of a time trend, a significant average decrease by 7% every second year was demonstrated. Lastly, a negative age gradient, with a PR of 0.48 (0.33–0.67) for the middle age group and PR 0.40 (0.26–0.59) for the youngest was found. Within the subgroup of hairdressers, the time trend was even more marked, with a decrease by 15% every two years. The negative age gradient was more pronounced than in the general analysis with a lesser risk in the middle age group of PR 0.49 (0.32–0.74) and especially in the youngest age group (PR 0.24 (0.13–0.41)). In consumers, no significant effect was noted except for a reduced risk in the middle age group only (PR 0.39 (0.19–0.73)).

## 4 Discussion

The present analysis evidently builds on the data used for the four successive similar analyses [14, 15, 16, 17], but, as a novel aspect, presents quantitative risk estimators for (i) long‐term time trends, (ii) the impact of belonging to the exposure subgroups of female hairdressers and consumers, respectively, and (iii) the risk associated with age group, based on log‐binomial regression modelling.

The previous analyses have helped to narrow down the scope of allergens considered to those who have been found important, either owing to the frequency of contact sensitization found or its implications. Consequently, a number of allergens who may well be of some importance, certainly in individual cases, have been omitted from the present analysis, such as resorcinol (a hitherto commonly used coupling agent for oxidative hair dyes, but a very rare allergen), formaldehyde and its liberators (owing to their limited and—in Europe—even decreasing importance as cosmetic product preservatives). Moreover, concerning fragrances, previously no clear association with hairdressing work and contact dermatitis related to (occupational exposure to) hair cosmetic products has been found [21]. In the following sections, some particular aspects will be discussed.

### 4.1 Regulatory Interventions and Their Effect

Is epidemiological surveillance based on clinical patch test data of patients with suspected allergic contact dermatitis able to identify trends, up‐ or downward, as a result of exposure changes in general, and of regulatory intervention in particular? The time trend results with glyceryl thioglocolate (GMTG) following drastic intervention in terms of a voluntary withdrawal by industry followed by a ban [22] clearly serve as a “positive control” for an affirmative response to this question. In this context, age‐stratification of time trend results allows the earliest possible recognition of a downward trend, because the youngest will not have been exposed, or just a minor part of them, depending on the age categorization chosen. Patch test results should generally be regarded as prevalences (among those patch tested, i.e., not directly related to the general population level) as it is unclear when sensitization actually occurred. However, observing a young subgroup over time, which has not been exposed after a certain preventive intervention can be regarded as an approximation at least of decreasing incidence.

Choosing age instead of seniority in the trade will be conservative, as some apprentices may have taken up work as hairdressers later in life and will be lost to the presumably non‐exposed subgroup of interest. In other instances, seniority may serve well to represent the beginning of (no) exposure to work materials, as demonstrated by a time trend analysis in construction workers who were already exposed before versus only after the introduction of ferrous sulphate to cement [23]. Nevertheless, some residual, perhaps low, exposure to GMTG is possible by products bought from outside Germany (GMTG is not restricted by EU law beyond the concentration limits also in place for salts of glycolic acid, that is 8% for hair products and 11% for depilatories, Annex III/2b to EC 1223/2009) [6]. The lack of regulation beyond Germany is interesting to note, as also elsewhere in Europe, GMTG is a frequent hair cosmetic allergen, and especially so in the USA, where 54% of hairdressers patch tested 1994–2010 were found sensitized [24]; however, since 2010, GMTG contact allergy seems to be declining [5], possibly related to fashion trends.

Another regulatory intervention, albeit with a much broader scope and impact, had been the withdrawal and later ban of methylisothiazolinone (MI) as preservative in leave‐on products in the EU, and a restriction to 15 ppm in‐product concentration in rinse‐off products following the unprecedented epidemic of MI contact allergy. The graph illustrates MCI/MI contact allergy in hairdressers and consumers, respectively (Figure S5). However, the driving force for the up‐ and downward trends of MCI/MI contact allergy—on a lower level than with MI—has been the in‐ and decrease of MI contact allergy [7, 25]. The general peak and subsequent decline to about a pre‐epidemic level of contact allergy is recognisable since around 2013/14 in both subgroups, with some “statistical noise” in the smallest subgroup of the youngest. Thus, both subgroups have benefited from the intervention, the consequences of which have been illustrated on a more general level, contrasted with the course given no intervention in the USA [7]. The even more interesting fact to note, however, is the higher prevalence of contact allergy to MCI/MI in hairdressers. This is also evident, in terms of an age‐adjusted analysis, from the regression results (Table 2). While it could be argued that hairdressers tend to be fashion‐sensitive and be using an above‐average amount of basically all cosmetic products, thereby increasing their risk to become sensitized by any (every‐day) cosmetic product, the increase in risk appears clearly in excess of anything expected for such putative above‐average self‐application.

Recognizing the respiratory sensitization potential of APS, use of dust‐free product formulations has been promoted, if just very partially followed [26]. However, this change in product formulation is not expected to decrease skin exposure, which needs to be avoided by wearing adequate protective gloves. Hence, the observed slight decrease of contact allergy in hairdressers (as well as consumers; Figure 5) seems to be related to possibly changing fashion habits—or better skin protection in the course of the study period.

### 4.2 Ingredients of Oxidative Hair Dyes and Cross‐Reactivity

First addressing single ingredients—before venturing to discuss cross‐reactivity as a contributory factor for observed sensitization frequencies and trends, respectively—it can be stated that during the study period, toluene‐2,5‐diamine (PTD) is the most important primary intermediate (in Germany [27], but possibly also in Switzerland and Austria as neighbouring and probably similar markets) – and certainly also in North America where PTD has been named “Allergen of the Year” for 2025 [28]. Conversely, *p*‐phenylenediamine (PPD) has at least on the German market been to a large extent replaced by PPD derivatives such as, *inter alia*, N,N‐bis(2‐hydroxyethyl)‐PPD sulfate, hydroxyethyl‐PPD sulfate [27], or 2‐methoxymethyl‐PPD [5], although the regulatory framework has not changed during the study period, stating that “After mixing under oxidative conditions the maximum concentration applied to hair must not exceed 2% calculated as free base” both for general and professional use, in EU Cosmetics Regulation, Annex III/8a [29]. The other primary intermediate, *p*‐aminophenol, has been found in around 21% of retail products and 36% of professionally used products, respectively, in the above‐mentioned German survey [27]. In some accordance with such presumably relatively broad exposure, sensitization is not uncommonly found, as illustrated in our analysis.

Other ingredients of oxidative hair dyes are termed secondary intermediates or, more colloquially, couplers. Upon oxidation through H_2_O_2_, couplers can selectively react with the oxidized primary intermediates, making them suitable to generate the final shade of hair colour within the hair shaft, thus, permanently [30]. As an example, *p*‐aminophenol acts much better as primary intermediate, while m‐aminophenol is the better coupler. *p*‐Aminophenol is more easily oxidized and directly forms quinoid structures due to its *para* positioning, allowing better resonance stabilization. By contrast, *m*‐aminophenol is more stable and oxidizes more slowly, achieving a more selective interaction with oxidized intermediates, thus being a better coupler for fine‐tuning of shades. Sensitization potency of *p*‐aminophenol and *m*‐aminophenol is different [31]. *p*‐Aminophenol is much more prone to form highly reactive structures, especially the quinone‐imine structure compared to *m*‐aminophenol. The formed reactive species (intermediates) of *p*‐aminophenol can form more and more stable adducts with cellular proteins, thereby increasing allergenicity. In contrast, *m*‐aminophenol forms fewer and less reactive protein complexes, lowering its allergenicity. In agreement, in our patch test data, *m*‐aminophenol is a much less frequent sensitizer than *p*‐aminophenol, PPD, PTD or other primary intermediates, and, again, more so in consumers than in hairdressers, and more so in younger patients (for a discussion of selection effects differing between these groups see section 4.3 below).

Couplers are rarely used alone, but usually in combination; *m*‐aminophenol is very commonly used, that is, in around 70% of consumer or professional products [27]. The biological and sensitizing activities of these substances are as heterogeneous as their chemical properties. In general, the commonly used primary intermediates PTD and *p*‐aminophenol are classified as strong sensitizers based on testing with toxicological animal models, though generally less potent than PPD. The modified derivative of PPD with a methoxymethyl group at the 2‐position of the benzene ring (*o*‐methoxymethyl‐PPD), which could not be considered in our analysis owing to lack of patch test data, exhibits significantly lower protein reactivity and dendritic cell activation than PPD and is classified as a moderate sensitizer [32].

In the case of hydroquinone, current exposure is unclear, especially in the youngest group with presumably no “historical” sensitization via use in oxidative hair dyes. In the Local Lymph Node Assay (LLNA), hydroquinone has been reported to be a strong sensitizer (EC_3_ ≈ 0.07%) [33]. The only permitted low hydroquinone exposure by cosmetics sold in the EU is by artificial nail systems, which may contain up to 0.02% hydroquinone after mixing (EU Cosmetics Regulation, Annex III/14) [6]. Also, sensitization through skin bleaching creams (which are easily accessible from vendors outside the EU via e‐commerce market places) or cross reactivity with arbutin and other skin‐bleaching agents are unlikely to play a role. The latter conclusion is based on testing the substances via the guinea pig maximization test (GPMT), demonstrating that glycosylation of hydroquinone (yielding arbutin) decreased the sensitization potency and cross‐reactivity to PPD compared to hydroquinone itself. Furthermore, no evidence of cross‐reactivity to arbutin in hydroquinone‐sensitized animals (0/4 animals in each of the 3 challenge dose groups) was found [33]. However, observed responses may still be based on cross‐reactivity. Hydroquinone is 1,4‐dihydroxybenzene and structurally closely related to PPD. Both show para‐substitutions; PPD has —NH_2_ at positions 1 and 4 (diamine) and hydroquinone —OH at 1 and 4. Also, other primary intermediates have *para*‐substituted aromatic structures, often with amino groups instead of hydroxyl groups. Even though —NH2 and OH are different groups, their position and ring structure are forming similarly structured intermediates that may to lead to cross‐reactivity (we offer possible structures as example in Figure S7) [34]. In line, patch test responses to hydroquinone were equally frequent in individuals responding to PPD and PTD. In addition, higher percentages were observed in individuals with a high degree of sensitization to PPD (+++ patch test scores compared to +, see Table 4).

As pyrogallol has been banned in 1992 in the EU, exposure (and origin of sensitization) especially of the youngest age group is unclear. Pyrogallol has three hydroxyl groups in ortho and meta positions and is structurally different from the other para‐substituted substances. Despite the structural differences, pyrogallol and PPD both undergo transformations, potentially leading to common reactive intermediates—thereby cross‐reactivity seems plausible [35]. Thus, especially in individuals with strong or broad aromatic amine contact sensitivity, pyrogallol may trigger reactions. In line with this notion, concomitant patch test reactivity between PPD (1% pet.) and pyrogallol was observed (Table 4) particularly in patients with a high degree of PPD sensitization.

In general, it is difficult to separate true immunological cross‐reactivity from multiple sensitization in a clinical setting, which may hamper identification of safe alternatives [36]. However, a predominance of concomitant reactions to primary para‐amino compounds (PAAs, e.g., PTD, *p*‐aminophenol, disperse (azo) dyes, (benzo)caine, *para*‐aminobenzoic acid (PABA) or black rubber components like *N*‐isopropyl‐*N*′‐phenyl‐p‐phenylenediamine (IPPD)) as well as to other hair dye ingredients which are chemically different from classical “PAA‐family” core substances but share similar intermediates during haptenization (e.g., *m*‐aminophenol, pyrogallol, Figure S7) were frequently reported among patients with positive patch test reactions to PPD [34, 37, 38]. In line with our study (Table 4), reactions to pyrogallol independent from PPD are not uncommon, although less frequent compared to PPD‐positive patients [34]. In another British study, concomitant reactions to Disperse Orange 3, which actually contains PPD, but not to caine mix III or IPPD were observed [39]. In such cases, asymmetric sensitization patterns have to be considered. The pattern of concomitant reactions seems to depend on the potency of the initially sensitizing compound and the degree of sensitization [40, 41, 42].

A valuable approach to investigate different patterns of concomitant patch test reactivity is the stratified analysis according to PPD patch test reaction strength. A high degree of sensitization usually occurs due to higher sensitizing dose/area and leads a low elicitation threshold. According to an IVDK study on different exposure sources known to be causative for PPD sensitization [43], hair dyeing is the major source among PPD positive patients. Moreover, temporary black henna tattoos, which were documented in a few patients only, were significantly associated with such a high degree of sensitization which usually leads to a pronounced pattern of concomitant patch test reactivity to the core PAA family substances [44] as well as other substances like pyrogallol, hydroquinones, *m*‐aminophenol or caines [43, 45, 46].

Taking a look at the youngest age groups, particularly children sensitized to black henna and without a history of exposure to permanent hair dyes, gives further insights. In a series of 4 cases with a history of henna tattooing, all 4 patients, of which 3 were paediatric patients, had extreme (+++) PPD reactions, and all reacted concomitantly to two or more textile dyes. Three of them also reacted to PTD and *p*‐aminophenol and 1 patient additionally reacted to *m*‐aminophenol, benzocaine, IPPD, hydroquinone, and pyrogallol [47]. As co‐exposure and co‐sensitization to such a variety of compounds seems improbable in these paediatric cases, cross‐reactivity is a probable explanation. Similarly, in a Danish study on paediatric patients sensitized to PPD by adulterated temporary black henna tattoos, similar responses to the core “PAA‐family” and beyond were reported: 3 of 8 children reacted to pyrogallol, 5/8 to hydroquinone, 6/8 to Disperse Orange 3, 7/8 to PTD, 7/8 to *m*‐aminophenol, and 8/8 to *p*‐aminophenol [48]. Moreover, 5 of those 8 also reacted to black rubber and caine mix, respectively, which are neither contained in hair dye nor in black henna preparations and where exposure in children can largely be excluded. Although pyrogallol [49] and particularly *m*‐aminophenol and *p*‐aminophenol can possibly be found in higher concentrations in commercial henna preparations [50], such re‐occurring patterns of concomitant patch test reactivity point to immunological cross reactivity mediated by common intermediates (see discussion above). The same applies to hydroquinone, which was, in addition to PPD and IPPD, found as accompanying allergen in a German report on a temporary black henna tattoo case [51]. However, confirmatory (in vitro) studies on cross reactivity on single T‐cell level are currently lacking [52].

### 4.3 Risk in Hairdressers and Consumers

As easily recognisable from the descriptive presentation, the share of consumers had always been greater and even increased over the 26 years of the study period. Does this indicate an increasing risk in consumers? Probably yes, see next paragraph. Does this imply a greater risk to become sensitized to hair cosmetic ingredients in consumers than in hairdressers? Certainly not, as the denominator of exposed consumers is much larger than the number of hairdressers (who may all be regarded as exposed, for simplification, although a few work only administratively, and about 15% (in Germany) only with very limited working hours): In Germany, the total labour force comprized some 240 000 hairdressers in 2020 [53]. An analysis of cases of occupational dermatitis notified in Northern Bavaria between 1990 and 1999, thus only partially overlapping with the initial study period, found an incidence of 97.4 cases per 100 000 hairdressers per year, which was the highest among all occupations [54]. In the meantime, during our study period, the incidence has dropped most likely thanks to improved primary and secondary prevention [55]. However, in view of the multiple exposures still challenging for hairdressers' skin, contact dermatitis, including allergic contact dermatitis, is still relatively frequent [2].

The number of “exposed” consumers, as denominator for the observed frequency of contact allergy to the hair cosmetic ingredients considered is more difficult to estimate. Probably everyone is exposed to shampoo, albeit perhaps once a day, compared to multiple exposures of the hairdresser's hands per day [1]. By contrast, specific products for permanent hair dyeing, bleaching or waving are used only by a certain part of the population. However, this proportion is large with regard to hair dyes: according to a computer‐assisted personal interview (CAPI) survey, permanent hair dyes were used by 2.45 million Germans (both male and female) at least monthly and by 9.67 million more rarely in 2017, with largely similar figures in 2020 [56]. Non‐permanent colouring was used somewhat more rarely, for example, by 1.82 million at least monthly and by 4 million more rarely, also comprising males and females, in 2017 [57]. Similar information has not been found regarding bleaching and waving/straightening products; however, these seem to be mostly applied in the salon, and not in home use, apparently entailing a much lesser risk in consumers than the widely used dyeing products. While these surveys did not distinguish between male and female users, the almost negligible proportion of male patients with suspected ACD to hair cosmetics among our patients (7%) seems to indicate that more females are exposed, and perhaps more intensely so, so that their risk of becoming sensitized is much higher than that of males.

Comparing the two subgroups, a lower level of prevalence to hair dye components in hairdressers compared to consumers could further be explained by the fact that hairdressers presenting with contact dermatitis, usually of their hands, are quite liberally patch tested not to overlook relevant contact sensitization, which could have important implications for secondary prevention. Thereby, sensitization may be “diluted” compared to consumers, who often do not consult a physician: According to a Norwegian survey, “only 17 percent of the women and 9% of the men who have experienced adverse effects have visited a medical practitioner because of this” [58]. Hence, those consumers who present in the secondary or tertiary care dermatological departments of the IVDK likely represent a more select, severely affected subset of consumers with hair cosmetic product‐related dermatitis—and correspondingly, a high share of (hitherto undetected) contact allergy and resulting allergic contact dermatitis.

### 4.4 Limitations

The present re‐analysis, as the four different original reports, relies on day (D)3 readings (or, substituting these when not performed, D4 readings). Thereby, a certain share of late positive reactions for example, first appearing (as positive) around D7 will be missed, depending on the allergen and on patient characteristics [59]. Unfortunately, evidence of the share of positive reactions potentially missed with regard to the present scope of allergens is lacking. Clinical experience with PAAs and, regarding PPD, a toxicokinetic study [60] seems to indicate that PAAs are probably leading to positive reactions quite fast with the patch test concentrations used.

### 4.5 Perspectives

In general, substitution of active hair cosmetic ingredients, notably primary and secondary intermediates, by less‐sensitizing alternatives still appears as a challenge, despite some PPD derivatives possibly showing lesser induction potential. In this situation, the adequate use of gloves suitable for the respective task is the most important preventive strategy. According to the results of a recent investigation from Denmark [61], hairdressers (including apprentices) seem to lack knowledge on how to use protective gloves correctly. However, a short demonstration of correct protective glove use made a significant difference [61]. This points to the fact that hairdressers urgently need to be trained on correct protective glove use, ideally during apprenticeship, and perhaps repeatedly. Additionally, protective gloves are being improved, for example, by using liners, semipermeable membranes or laminates to increase wearing comfort and decrease epidermal barrier perturbation by occlusion [62].

## 5 Conclusions

The results presented illustrate several aspects:

- Rigorous regulation, as in case of GMTG or MI, usually triggered only by comparatively massive contact allergy problems, shows clearly visible effects, especially if the youngest subgroup of patch tested patients is looked at, in whom the share of possibly “historical”, pre‐intervention sensitization is low or non‐existent;
- In case of marked cross‐reactivity between compounds, it is difficult to discern the effect of intervention, as, in our results, the ban of pyrogallol in 1992 (owing to mutagenicity concerns);
- Other, non‐cosmetic sources of exposure may interfere with a clearly recognisable time trend, e.g., in case of hydroquinone, which has been banned in hair dyes, but is probably still in use in skin‐bleaching (prescription) topical preparations;
- Although the absolute risk of contact allergy to all agents examined is estimated to be much higher in hairdressers, the increase in (i) patch tested and (ii) positively tested consumers, especially young ones, is of concern.

## Author Contributions

**Wolfgang Uter:** conceptualization, methodology, software, data curation, formal analysis, visualization, writing – original draft, writing – review and editing. **Jakob Ferløv Baselius Schwensen:** validation, writing – original draft, Writing – review and editing, conceptualization. **Brunhilde Blömeke:** validation, writing – review and editing, methodology. **Olaf Gefeller:** methodology, visualization, supervision, writing – original draft, writing – review and editing. **Swen M. John:** writing – original draft, writing – review and editing. **Cara Bieck:** writing – original draft, writing – review and editing. **Steffen Schubert:** funding acquisition, data curation, software, methodology, validation, project administration, writing – original draft, writing – review and editing.

## Conflicts of Interest

W. Uter has accepted research funds for the department from the IDEA project (IFRA, https://ifrafragrance.org/). J.F.B. Schwensen has served as investigator for Sanofi Aventis and speaker for Galderma. The IVDK, maintained by the IVDK e.V., of which S. Schubert is an employee, is sponsored by, *inter alia*, the cosmetic and fragrance industry (associations). The other authors declare no conflicts of interest.

## Supporting information


**Table S1:** The versions of the DKG hair cosmetic series used during the study period. All allergens in water, except where indicated otherwise: ^a^, aqua.
**Table S2:** Population characteristics of both subgroups, female hairdressers and consumers, as tested in the IVDK between 01/1995 and 12/2020.
**Table S3:** Patch test results with primary intermediates of oxidative hair dyes included in the “Hairdresser Series” (in different versions, hence variable testing across the periods) in female hairdressers (*n* = 2678) and consumers (*n* = 6244), resp., patch tested 1995–2020 in the departments of the IVDK. %pos (std.), age‐stratified prevalence with accompanying 95% exact confidence interval (CI). (A) toluene‐2,5 diamine 1% pet. (syn. *p*‐toluylenediamine, PTD); (B) *p*‐phenylenediamine 1% pet. (PPD, tested in the baseline series, in this concentration, until 2004); C *p*‐aminophenol 1% pet.
**Table S4:** Patch test results with secondary intermediates of oxidative hair dyes included in the “Hairdresser Series” (in different versions, hence variable testing across the periods) in female hairdressers (*n* = 2678) and consumers (*n* = 6244), resp., patch tested 1995–2020 in the departments of the IVDK. %pos(std.), age‐stratified prevalence with accompanying 95% exact confidence interval (CI). (A) *m*‐aminophenol 1% pet. (syn. 3‐aminophenol); (B) hydroquinone 1% pet.; C pyrogallol 1% pet.
**Table S5:** Patch test results with the bleaching agent ammonium persulfate 2.5% pet., included in the “Hairdresser Series”, observed in female hairdressers (*n* = 2678) and consumers (*n* = 6244), resp., patch tested 1995–2020 in the departments of the IVDK. %pos(std.), age‐stratified prevalence with accompanying 95% exact confidence interval (CI).
**Table S6:** Patch test results with the waving/relaxing agents included in the “Hairdresser Series”, observed in female hairdressers (*n* = 2678) and consumers (*n* = 6244), resp., patch tested 1995–2020 in the departments of the IVDK. %pos(std.), age‐stratified prevalence with accompanying 95% exact confidence interval (CI). (A) ammonium thioglycolate (ATG) 1% aqu.; (B) glyceryl thioglycolate (GMTG) 1% pet.
**Table S7:** Patch test results with two preservatives included in the “Baseline Series”, observed in female hairdressers (*n* = 2678) and consumers (*n* = 6244), resp., patch tested 1995–2020 in the departments of the IVDK. %pos(std.), age‐stratified prevalence with accompanying 95% exact confidence interval (CI). (A) Methylchloroisothiazolinone/methylisothiazolinone (MCI/MI) 0.01% aqu.; (B) methyldibromo glutaronitrile (MDBGN) 0.2% pet. (1997–2015) and 0.3% pet. (since 2016), respectively.

**Figure S1:** The share of hairdressers and clients, respectively, among all patch tested female patients consulting the departments of the IVDK between 1995 and 2020.

**Figure S2:** Time trend of positive patch test reactions (crude, age‐stratified prevalences) to *p*‐phenylenediamine (PPD) 1% pet. in female hairdressers (left) and female consumers (right) consulting the departments of the IVDK between 1995 and 2020. As this allergen has not been regularly tested during 5 years, between 01/2005 and 12/2009, these years were excluded in the graph.

**Figure S3:** Time trend of positive patch test reactions (crude, age‐stratified prevalences) to pyrogallol 1% pet. in female hairdressers (left) and female consumers (right) consulting the departments of the IVDK between 1995 and 2020.

**Figure S4:** Time trend of positive patch test reactions (crude, age‐stratified prevalences) to ammonium thioglycolate (ATG) 1% aqu. in female hairdressers (left) and female consumers (right) consulting the departments of the IVDK between 2004 and 2020.

**Figure S5:** Time trend of positive patch test reactions (crude, age‐stratified prevalences) to methylchloroisothiazolinone/methylisothiazolinone 3:1 (MCI/MI) 0.01% aqu. in female hairdressers (left) and female consumers (right) consulting the departments of the IVDK between 2004 and 2020.

**Figure S6:** Time trend of positive patch test reactions (crude, age‐stratified prevalences) to methyldibromo glutaronitrile (MDBGN) 0.2 or 0.3% pet. (see text in “results”) in female hairdressers (left) and female consumers (right) consulting the departments of the IVDK between 2004 and 2020.

**Figure S7:** Transformation of structurally related and unrelated chemicals: examples of possible formation of benzoquinone or benzoquinone derivatives.

## Data Availability

The data that support the findings of this study are not publicly available due to privacy or ethical restrictions.
