# Supplementary material for: Contact Allergy to Ingredients of Hair Cosmetics Associated with Occupational and Non‐Occupational Exposure—Trends from 1995 to 2020 in Central Europe, with or without Regulation
Source: Contact Dermatitis. 2025 Dec 30;94(4):347–63. doi: 10.1111/cod.70079 (PMC12956424; doi:10.1111/cod.70079)
Supplement: Supplementary file 1 — Table S1: The versions of the DKG hair cosmetic series used during the study period. All allergens in water, except where indicated otherwise: a, aqua. Table S2: Population characteristics of both subgroups, female hairdressers and consumers, as tested in the IVDK between 01/1995 and 12/2020. Table S3: Patch test results with primary intermediates of oxidative hair dyes included in the “Hairdresser Series” (in different versions, hence variable testing across the periods) in female hairdressers (n = 2678) and consumers (n = 6244), resp., patch tested 1995–2020 in the departments of the IVDK. %pos (std.), age‐stratified prevalence with accompanying 95% exact confidence interval (CI). (A) toluene‐2,5 diamine 1% pet. (syn. p‐toluylenediamine, PTD); (B) p‐phenylenediamine 1% pet. (PPD, tested in the baseline series, in this concentration, until 2004); C p‐aminophenol 1% pet. Table S4: Patch test results with secondary intermediates of oxidative hair dyes included in the “Hairdresser Series” (in different versions, hence variable testing across the periods) in female hairdressers (n = 2678) and consumers (n = 6244), resp., patch tested 1995–2020 in the departments of the IVDK. %pos(std.), age‐stratified prevalence with accompanying 95% exact confidence interval (CI). (A) m‐aminophenol 1% pet. (syn. 3‐aminophenol); (B) hydroquinone 1% pet.; C pyrogallol 1% pet. Table S5: Patch test results with the bleaching agent ammonium persulfate 2.5% pet., included in the “Hairdresser Series”, observed in female hairdressers (n = 2678) and consumers (n = 6244), resp., patch tested 1995–2020 in the departments of the IVDK. %pos(std.), age‐stratified prevalence with accompanying 95% exact confidence interval (CI). Table S6: Patch test results with the waving/relaxing agents included in the “Hairdresser Series”, observed in female hairdressers (n = 2678) and consumers (n = 6244), resp., patch tested 1995–2020 in the departments of the IVDK. %pos(std.), age‐stratified prevalence with acc [file COD-94-347-s008.docx]

**Online supplemental table S1:** The versions of the DKG hair cosmetic series used during the study period. All allergens in water, except where indicated otherwise: ^a^, aqua

| Allergen | Period included |
| --- | --- |
| Toluene-2,5-diamine 1% pet. | 01/1992-12/2020 |
| *p*-Phenylenediamine 1% pet.^a)^ | 01/2010-12/2020 |
| Ammonium persulfate 2.5% pet. | 01/1992-12/2020 |
| 4-Amino-2-hydroxytoluene 1% pet. | 10/2016-12/2020 |
| *p*-Aminophenol 1% pet. | 01/1992-12/2020 |
| Glyceryl thioglycolate 1% pet. | 01/1992-12/2020 |
| *m*-Aminophenol 1% pet. | 01/1992-12/2020 |
| Decyl glucoside 5% pet. | 10/2016-12/2020 |
| Hydroxyethyl-*p*-phenylendiamine sulfate 2% pet. | 10/2016-12/2020 |
| Ammonium thioglycolate 1% pet. | 01/1992-11/2002 |
| Ammonium thioglycolate 1% aqu. | 01/2004-12/2020 |
| Lauryl glucoside 3% pet. | 10/2016-12/2020 |
| Hydroquinone 1% pet. | 01/1992-08/2015 |
| Pyrogallol 1% pet. | 01/1992-06/2019 |
| Ethanolamine 2% pet. | 11/2002-12/2020 |
| Cysteamine HCl 0.5% pet. | 10/2016-12/2020 |
| 2-Methylresorcinol 1% pet. | 10/2016-12/2020 |
| *p*-Methylaminophenol sulfate 1% pet. | 01/2015-12/2020 |
| Resorcinol 1% pet. | 09/2015-12/2020 |
| Cocamidopropyl betaine 1% aqu. | 07/2004-12/2020 |

^a)^ PPD had been tested in the baseline series until 12/2004, which was also considered in this analysis.

**Online supplemental table S2:** Population characteristics of both subgroups, female hairdressers and consumers, as tested in the IVDK between 01/1995 and 12/2020.

| Period | Hairdressers |  |  |  | Consumers |  |  |  | Age | (median) |
| --- | --- | --- | --- | --- | --- | --- | --- | --- | --- | --- |
|  | AT | CH | DE | total | AT | CH | DE | total | Hairdresser | Consumers |
| 1995/96 | 37 | 0 | 157 | 194 | 47 | 0 | 202 | 249 | 24 | 44 |
| 1997/98 | 23 | 0 | 174 | 197 | 52 | 0 | 313 | 365 | 23 | 47 |
| 1999/2000 | 17 | 0 | 171 | 188 | 41 | 0 | 206 | 247 | 24 | 44 |
| 2001/02 | 23 | 3 | 184 | 210 | 31 | 4 | 249 | 284 | 22.5 | 47 |
| 2003/04 | 17 | 2 | 190 | 209 | 31 | 21 | 351 | 403 | 23 | 49 |
| 2005/06 | 10 | 4 | 148 | 162 | 27 | 15 | 346 | 388 | 26 | 50 |
| 2007/08 | 12 | 42 | 198 | 252 | 31 | 78 | 535 | 644 | 22.5 | 50 |
| 2009/10 | 12 | 28 | 240 | 280 | 28 | 78 | 642 | 748 | 23 | 50 |
| 2011/12 | 15 | 36 | 263 | 314 | 45 | 87 | 609 | 741 | 27 | 49 |
| 2013/14 | 16 | 49 | 236 | 301 | 33 | 92 | 579 | 704 | 28 | 48 |
| 2015/16 | 11 | 26 | 167 | 204 | 22 | 86 | 547 | 655 | 26 | 49 |
| 2017/18 | 25 | 22 | 163 | 210 | 29 | 101 | 422 | 552 | 29.5 | 50 |
| 2019/20 | 41 | 24 | 140 | 205 | 24 | 82 | 304 | 410 | 29 | 49 |

AT, Austria; CH, Switzerland; DE, Germany

**Online supplemental table S3:** Patch test results with primary intermediates of oxidative hair dyes included in the “Hairdresser Series” (in different versions, hence variable testing across the periods) in female hairdressers (n=2678) and consumers (n=6244), resp., patch tested 1995–2020 in the departments of the IVDK. %pos(std.), age-stratified prevalence with accompanying 95% exact confidence interval (CI). **(A)** toluene-2,5 diamine 1% pet. (syn. *p*-toluylenediamine, PTD); **(B)** *p*-phenylenediamine 1% pet. (PPD, tested in the baseline series, in this concentration, until 2004); **C** *p*-aminophenol 1% pet.

**(A) PTD**

| Period | n(test) | $\leq$ 20 y | 21–32 y | $>$ 32 y | $\leq$ 20 y | 21–32 y | $>$ 32 y |
| --- | --- | --- | --- | --- | --- | --- | --- |
|  |  | Hairdressers |  |  | Consumers |  |  |
| 1995-96 | 375 | 28.9% (19.1–40.5%) | 21% (11.7–33.2%) | 15% (5.7–29.8%) | 13.3% (3.8–30.7%) | 17.6% (6.8–34.5%) | 6.3% (2.8–12.1%) |
| 1997-98 | 455 | 27% (16.6–39.7%) | 13.7% (5.7–26.3%) | 15.9% (6.6–30.1%) | 6.9% (0.8–22.8%) | 14.5% (6.5–26.7%) | 7.7% (4.5–12.2%) |
| 1999-2000 | 372 | 18.6% (9.7–30.9%) | 14% (5.8–26.7%) | 15.3% (7.2–27%) | 7.7% (0.9–25.1%) | 30.3% (15.6–48.7%) | 16.1% (10.5–23.1%) |
| 2001-02 | 406 | 22.1% (12.9–33.8%) | 29.8% (18.4–43.4%) | 10.2% (3.8–20.8%) | 20.8% (7.1–42.2%) | 16.7% (5.6–34.7%) | 15.7% (10.5–22.1%) |
| 2003-04 | 499 | 21.4% (12.5–32.9%) | 11.3% (4.3–23%) | 14.5% (6.9–25.8%) | 37.8% (22.5–55.2%) | 38.9% (23.1–56.5%) | 14.6% (10.4–19.7%) |
| 2005-06 | 411 | 19.4% (8.2–36%) | 29.3% (16.1–45.5%) | 16.3% (7.3–29.7%) | 50% (28.2–71.8%) | 32.4% (17.4–50.5%) | 15.8% (11.3–21.2%) |
| 2007-08 | 706 | 38.7% (27.6–50.6%) | 17.8% (9.8–28.5%) | 18.5% (9.3–31.4%) | 43.8% (29.5–58.8%) | 32.8% (21.6–45.7%) | 16.1% (12.6–20.1%) |
| 2009-10 | 868 | 22.7% (13.8–33.8%) | 19.1% (11.8–28.6%) | 17.7% (9.2–29.5%) | 67.3% (52.9–79.7%) | 24.7% (15.6–35.8%) | 17% (13.8–20.5%) |
| 2011-12 | 831 | 21.9% (9.3–40%) | 20.5% (13.7–28.7%) | 19% (11.3–29.1%) | 57.8% (42.2–72.3%) | 31.3% (21.6–42.4%) | 20.9% (17.3–24.8%) |
| 2013-14 | 833 | 27% (16.6–39.7%) | 20.7% (12.9–30.4%) | 24.2% (16.2–33.9%) | 66% (51.2–78.8%) | 32.1% (22.2–43.4%) | 25.9% (21.9–30.2%) |
| 2015-16 | 732 | 26.2% (13.9–42%) | 12.1% (5.4–22.5%) | 17.2% (8.6–29.4%) | 53.3% (34.3–71.7%) | 45.5% (34.1–57.2%) | 23.7% (19.9–27.9%) |
| 2017-18 | 635 | 29.3% (16.1–45.5%) | 8.1% (2.7–17.8%) | 23.9% (14.3–35.9%) | 71.4% (51.3–86.8%) | 33.3% (21.7–46.7%) | 21.5% (17.5–26%) |
| 2019-20 | 509 | 25.6% (13–42.1%) | 5.7% (1.2–15.7%) | 24.2% (14.2–36.7%) | 56.7% (37.4–74.5%) | 34.9% (21–50.9%) | 16% (11.9–20.8%) |

**(B) PPD** (2005 to 2008: rare, irregular testing of PPD 1% pet., hence, results not shown)

| Period | n(test) | $\leq$ 20 y | 21–32 y | $>$ 32 y | $\leq$ 20 y | 21–32 y | $>$ 32 y |
| --- | --- | --- | --- | --- | --- | --- | --- |
|  |  | Hairdressers |  |  | Consumers |  |  |
| 1995-96 | 424 | 27.6% (18–39.1%) | 26.6% (16.3–39.1%) | 7.3% (1.5–19.9%) | 15.6% (5.3–32.8%) | 20% (9.6–34.6%) | 11.3% (6.8–17.3%) |
| 1997-98 | 500 | 18.3% (9.5–30.4%) | 10.4% (3.5–22.7%) | 20% (9.6–34.6%) | 6.1% (0.7–20.2%) | 20% (11.1–31.8%) | 9.1% (5.8–13.4%) |
| 1999-2000 | 397 | 23.7% (13.6–36.6%) | 16% (7.2–29.1%) | 10.9% (4.1–22.2%) | 11.5% (2.4–30.2%) | 32.4% (18–49.8%) | 15.5% (10.4–21.8%) |
| 2001-02 | 422 | 14.5% (6.9–25.8%) | 17.6% (8.4–30.9%) | 3.4% (0.4–11.9%) | 20.7% (8–39.7%) | 18.2% (7–35.5%) | 15.1% (10.2–21%) |
| 2003-04 | 515 | 12.3% (5.5–22.8%) | 16% (7.2–29.1%) | 12.9% (5.7–23.9%) | 34.3% (19.1–52.2%) | 28.9% (16.4–44.3%) | 14% (10–18.8%) |
| 2005-06 |  |  |  |  |  |  |  |
| 2007-08 |  |  |  |  |  |  |  |
| 2009-10 | 490 | 18.2% (8.2–32.7%) | 22.4% (12.5–35.3%) | 24.4% (12.4–40.3%) | 62.1% (42.3–79.3%) | 17.9% (7.5–33.5%) | 20.8% (16.2–26%) |
| 2011-12 | 838 | 16.1% (5.5–33.7%) | 18.7% (12.2–26.7%) | 20.5% (12.6–30.4%) | 54.5% (38.8–69.6%) | 30.5% (20.8–41.6%) | 20.5% (16.9–24.4%) |
| 2013-14 | 836 | 20.6% (11.5–32.7%) | 19.8% (12.2–29.4%) | 14.9% (8.4–23.7%) | 61.2% (46.2–74.8%) | 32.9% (23.1–44%) | 26% (22–30.3%) |
| 2015-16 | 744 | 25.6% (13.5–41.2%) | 22.1% (12.9–33.8%) | 18.6% (9.7–30.9%) | 55.2% (35.7–73.6%) | 43.2% (32.2–54.7%) | 24.8% (20.9–29%) |
| 2017-18 | 645 | 29.3% (16.1–45.5%) | 12.7% (5.6–23.5%) | 15.2% (7.5–26.1%) | 75% (55.1–89.3%) | 31.1% (19.9–44.3%) | 22.3% (18.3–26.8%) |
| 2019-20 | 522 | 26.8% (14.2–42.9%) | 15.1% (6.7–27.6%) | 31.7% (20.6–44.7%) | 57.1% (37.2–75.5%) | 44.4% (29.6–60%) | 22.6% (17.9–27.8%) |

**(C) *p*-Aminophenol**

| Period | n(test) | $\leq$ 20 y | 21–32 y | $>$ 32 y | $\leq$ 20 y | 21–32 y | $>$ 32 y |
| --- | --- | --- | --- | --- | --- | --- | --- |
|  |  | Hairdressers |  |  | Consumers |  |  |
| 1995-96 | 369 | 9.2% (3.8–18.1%) | 4.8% (1–13.3%) | 0% (0–8.8%) | 3.6% (0.1–18.3%) | 9.4% (2–25%) | 2.4% (0.5–7%) |
| 1997-98 | 458 | 3.1% (0.4–10.8%) | 3.8% (0.5–13.2%) | 0% (0–7.9%) | 0% (0–11.9%) | 7.3% (2–17.6%) | 4.3% (2–8.1%) |
| 1999-2000 | 373 | 3.4% (0.4–11.7%) | 3.9% (0.5–13.5%) | 3.4% (0.4–11.7%) | 7.7% (0.9–25.1%) | 21.2% (9–38.9%) | 6.3% (2.9–11.6%) |
| 2001-02 | 407 | 1.5% (0–7.9%) | 7.1% (2–17.3%) | 3.4% (0.4–11.7%) | 16.7% (4.7–37.4%) | 12.9% (3.6–29.8%) | 5.4% (2.5–10%) |
| 2003-04 | 501 | 7.1% (2.4–15.9%) | 3.8% (0.5–13%) | 0% (0–5.9%) | 31.6% (17.5–48.7%) | 15.8% (6–31.3%) | 2.5% (0.9–5.3%) |
| 2005-06 | 418 | 5.4% (0.7–18.2%) | 11.9% (4–25.6%) | 3.9% (0.5–13.5%) | 43.5% (23.2–65.5%) | 29.4% (15.1–47.5%) | 4.8% (2.4–8.4%) |
| 2007-08 | 712 | 11.8% (5.6–21.3%) | 4.1% (0.8–11.4%) | 3.6% (0.4–12.5%) | 43.8% (29.5–58.8%) | 16.9% (8.8–28.3%) | 7.9% (5.4–11%) |
| 2009-10 | 868 | 4.1% (0.8–11.4%) | 1.1% (0–5.7%) | 3.2% (0.4–11.2%) | 63.5% (49–76.4%) | 11.8% (5.6–21.3%) | 5.7% (3.9–8.1%) |
| 2011-12 | 836 | 3.1% (0.1–16.2%) | 4.1% (1.3–9.3%) | 5.8% (1.9–13%) | 51.1% (35.8–66.3%) | 23.8% (15.2–34.3%) | 6.4% (4.4–9%) |
| 2013-14 | 833 | 6.3% (1.8–15.5%) | 5.6% (1.8–12.5%) | 4% (1.1–10%) | 56% (41.3–70%) | 25.6% (16.6–36.4%) | 5.8% (3.8–8.4%) |
| 2015-16 | 734 | 9.3% (2.6–22.1%) | 3% (0.4–10.4%) | 5.2% (1.1–14.4%) | 43.3% (25.5–62.6%) | 29.9% (20–41.4%) | 9.2% (6.7–12.2%) |
| 2017-18 | 640 | 7.3% (1.5–19.9%) | 3.1% (0.4–10.8%) | 3% (0.4–10.4%) | 72.4% (52.8–87.3%) | 15.3% (7.2–27%) | 7.7% (5.2–10.8%) |
| 2019-20 | 528 | 7.3% (1.5–19.9%) | 1.9% (0–9.9%) | 5.9% (1.6–14.4%) | 60% (40.6–77.3%) | 31.1% (18.2–46.6%) | 7.6% (4.8–11.3%) |

**Online supplemental table S4:** Patch test results with secondary intermediates of oxidative hair dyes included in the “Hairdresser Series” (in different versions, hence variable testing across the periods) in female hairdressers (n=2678) and consumers (n=6244), resp., patch tested 1995–2020 in the departments of the IVDK. %pos(std.), age-stratified prevalence with accompanying 95% exact confidence interval (CI). **(A)** *m*-aminophenol 1% pet. (syn. 3-aminophenol); **(B)** hydroquinone 1% pet.; **C** pyrogallol 1% pet.

**(A) *m*-Aminophenol**

| Period | n(test) | $\leq$ 20 y | 21–32 y | $>$ 32 y | $\leq$ 20 y | 21–32 y | $>$ 32 y |
| --- | --- | --- | --- | --- | --- | --- | --- |
|  |  | Hairdressers |  |  | Consumers |  |  |
| 1995-96 | 367 | 2.7% (0.3–9.3%) | 1.6% (0–8.5%) | 0% (0–8.8%) | 3.6% (0.1–18.3%) | 6.2% (0.8–20.8%) | 2.5% (0.5–7%) |
| 1997-98 | 455 | 1.6% (0–8.5%) | 0% (0–7%) | 2.2% (0.1–11.8%) | 0% (0–11.9%) | 3.6% (0.4–12.5%) | 2.9% (1.1–6.2%) |
| 1999-2000 | 372 | 3.4% (0.4–11.7%) | 3.9% (0.5–13.5%) | 1.7% (0–9.1%) | 0% (0–13.2%) | 15.2% (5.1–31.9%) | 4.9% (2–9.9%) |
| 2001-02 | 406 | 0% (0–5.3%) | 5.4% (1.1–14.9%) | 0% (0–6.1%) | 12.5% (2.7–32.4%) | 0% (0–11.2%) | 3% (1–6.9%) |
| 2003-04 | 500 | 1.4% (0–7.7%) | 3.8% (0.5–13%) | 0% (0–5.9%) | 26.3% (13.4–43.1%) | 15.8% (6–31.3%) | 0.8% (0.1–3%) |
| 2005-06 | 419 | 0% (0–9.5%) | 2.4% (0.1–12.6%) | 3.9% (0.5–13.5%) | 32% (14.9–53.5%) | 21.2% (9–38.9%) | 3% (1.2–6.2%) |
| 2007-08 | 712 | 5.3% (1.5–12.9%) | 0% (0–4.9%) | 3.6% (0.4–12.5%) | 30.6% (18.3–45.4%) | 10.8% (4.4–20.9%) | 5.1% (3.1–7.8%) |
| 2009-10 | 859 | 5.5% (1.5–13.4%) | 1.1% (0–5.8%) | 1.6% (0–8.5%) | 51.9% (37.6–66%) | 6.6% (2.2–14.7%) | 3% (1.7–4.9%) |
| 2011-12 | 831 | 3.1% (0.1–16.2%) | 3.3% (0.9–8.2%) | 2.4% (0.3–8.2%) | 28.9% (16.4–44.3%) | 17.9% (10.4–27.7%) | 4.3% (2.7–6.6%) |
| 2013-14 | 834 | 3.2% (0.4–11%) | 3.3% (0.7–9.3%) | 5.1% (1.7–11.4%) | 48% (33.7–62.6%) | 19.5% (11.6–29.7%) | 4.7% (2.9–7.1%) |
| 2015-16 | 736 | 0% (0–8.2%) | 0% (0–5.4%) | 1.7% (0–9.2%) | 23.3% (9.9–42.3%) | 21.8% (13.2–32.6%) | 6.1% (4.1–8.7%) |
| 2017-18 | 639 | 4.9% (0.6–16.5%) | 4.8% (1–13.5%) | 3% (0.4–10.4%) | 62.1% (42.3–79.3%) | 15% (7.1–26.6%) | 5.8% (3.7–8.7%) |
| 2019-20 | 536 | 2.4% (0.1–12.6%) | 1.8% (0–9.6%) | 4.3% (0.9–12.2%) | 38.7% (21.8–57.8%) | 19.6% (9.4–33.9%) | 3.8% (1.9–6.6%) |

**(B) Hydroquinone**

| Period | n(test) | $\leq$ 20 y | 21–32 y | $>$ 32 y | $\leq$ 20 y | 21–32 y | $>$ 32 y |
| --- | --- | --- | --- | --- | --- | --- | --- |
|  |  | Hairdressers |  |  | Consumers |  |  |
| 1995-96 | 372 | 0% (0–4.8%) | 0% (0–5.6%) | 0% (0–8.4%) | 7.1% (0.9–23.5%) | 0% (0–10.9%) | 0.8% (0–4.4%) |
| 1997-98 | 463 | 0% (0–5.5%) | 0% (0–7%) | 0% (0–7.5%) | 0% (0–11.9%) | 0% (0–6.4%) | 1% (0.1–3.4%) |
| 1999-2000 | 381 | 0% (0–5.9%) | 1.9% (0–10.3%) | 0% (0–6.1%) | 0% (0–13.2%) | 2.9% (0.1–14.9%) | 0% (0–2.5%) |
| 2001-02 | 410 | 0% (0–5.3%) | 1.8% (0–9.4%) | 0% (0–6.1%) | 4.2% (0.1–21.1%) | 3% (0.1–15.8%) | 1.8% (0.4–5.2%) |
| 2003-04 | 500 | 2.9% (0.3–9.9%) | 0% (0–6.8%) | 1.6% (0–8.7%) | 23.7% (11.4–40.2%) | 2.6% (0.1–13.8%) | 0.4% (0–2.3%) |
| 2005-06 | 419 | 0% (0–9.5%) | 0% (0–8.4%) | 2% (0–10.4%) | 24% (9.4–45.1%) | 15.2% (5.1–31.9%) | 0.4% (0–2.4%) |
| 2007-08 | 713 | 0% (0–4.7%) | 1.4% (0–7.3%) | 0% (0–6.5%) | 27.1% (15.3–41.8%) | 6.2% (1.7–15%) | 2% (0.9–4%) |
| 2009-10 | 868 | 1.4% (0–7.3%) | 0% (0–3.9%) | 0% (0–5.5%) | 40.4% (27–54.9%) | 5.3% (1.5–12.9%) | 0.4% (0–1.4%) |
| 2011-12 | 836 | 0% (0–10.9%) | 0.8% (0–4.6%) | 0% (0–4.2%) | 28.3% (16–43.5%) | 5.9% (1.9–13.2%) | 0.2% (0–1.2%) |
| 2013-14 | 841 | 3.2% (0.4–11%) | 1.1% (0–5.9%) | 0% (0–3.7%) | 32% (19.5–46.7%) | 7.2% (2.7–15.1%) | 0.2% (0–1.2%) |

**(C) Pyrogallol**

| Period | n(test) | $\leq$ 20 y | 21–32 y | $>$ 32 y | $\leq$ 20 y | 21–32 y | $>$ 32 y |
| --- | --- | --- | --- | --- | --- | --- | --- |
|  |  | Hairdressers |  |  | Consumers |  |  |
| 1995-96 | 367 | 4% (0.8–11.2%) | 9.4% (3.5–19.3%) | 5% (0.6–16.9%) | 3.6% (0.1–18.3%) | 9.4% (2–25%) | 1.7% (0.2–5.8%) |
| 1997-98 | 454 | 7.8% (2.6–17.3%) | 8.2% (2.3–19.6%) | 4.4% (0.5–15.1%) | 6.9% (0.8–22.8%) | 7.3% (2–17.6%) | 2.4% (0.8–5.6%) |
| 1999-2000 | 367 | 6.8% (1.9–16.5%) | 10% (3.3–21.8%) | 3.5% (0.4–12.1%) | 12.5% (2.7–32.4%) | 3% (0.1–15.8%) | 6.3% (2.9–11.7%) |
| 2001-02 | 407 | 1.4% (0–7.8%) | 0% (0–6.4%) | 0% (0–6.1%) | 0% (0–14.2%) | 6.5% (0.8–21.4%) | 4.2% (1.7–8.5%) |
| 2003-04 | 500 | 5.7% (1.6–14%) | 7.7% (2.1–18.5%) | 1.6% (0–8.7%) | 21.1% (9.6–37.3%) | 5.3% (0.6–17.7%) | 4.2% (2–7.5%) |
| 2005-06 | 421 | 2.7% (0.1–14.2%) | 7% (1.5–19.1%) | 2% (0–10.4%) | 23.1% (9–43.6%) | 18.2% (7–35.5%) | 1.7% (0.5–4.4%) |
| 2007-08 | 707 | 9.2% (3.8–18.1%) | 2.8% (0.3–9.7%) | 1.9% (0–9.9%) | 20.8% (10.5–35%) | 4.6% (1–12.9%) | 3.1% (1.6–5.3%) |
| 2009-10 | 863 | 1.3% (0–7.1%) | 3.1% (0.6–8.9%) | 1.6% (0–8.7%) | 17.3% (8.2–30.3%) | 7.9% (3–16.4%) | 2.6% (1.4–4.4%) |
| 2011-12 | 842 | 0% (0–10.6%) | 2.4% (0.5–7%) | 1.1% (0–6.2%) | 8.7% (2.4–20.8%) | 8.2% (3.4–16.2%) | 0.6% (0.1–1.9%) |
| 2013-14 | 839 | 1.6% (0–8.5%) | 3.2% (0.7–9.1%) | 2% (0.2–6.9%) | 20% (10–33.7%) | 4.8% (1.3–11.9%) | 0.7% (0.1–1.9%) |
| 2015-16 | 738 | 4.7% (0.6–15.8%) | 4.5% (0.9–12.5%) | 0% (0–6.1%) | 10% (2.1–26.5%) | 5% (1.4–12.3%) | 1.7% (0.8–3.4%) |
| 2017-18 | 646 | 2.4% (0.1–12.6%) | 0% (0–5.6%) | 1.4% (0–7.7%) | 16.7% (5.6–34.7%) | 0% (0–6.1%) | 1.1% (0.3–2.7%) |

**Online supplemental table S5:** Patch test results with the bleaching agent ammonium persulfate 2.5% pet., included in the “Hairdresser Series”, observed in female hairdressers (n=2678) and consumers (n=6244), resp., patch tested 1995–2020 in the departments of the IVDK. %pos(std.), age-stratified prevalence with accompanying 95% exact confidence interval (CI).

| Period | n(test) | $\leq$ 20 y | 21–32 y | $>$ 32 y | $\leq$ 20 y | 21–32 y | $>$ 32 y |
| --- | --- | --- | --- | --- | --- | --- | --- |
|  |  | Hairdressers |  |  | Consumers |  |  |
| 1995-96 | 365 | 36.5% (25.6–48.5%) | 7.9% (2.6–17.6%) | 10.3% (2.9–24.2%) | 7.1% (0.9–23.5%) | 6.2% (0.8–20.8%) | 1.6% (0.2–5.8%) |
| 1997-98 | 456 | 46.2% (33.7–59%) | 14% (5.8–26.7%) | 15.6% (6.5–29.5%) | 0% (0–11.9%) | 0% (0–6.5%) | 2.9% (1.1–6.2%) |
| 1999-2000 | 368 | 33.9% (21.8–47.8%) | 19.6% (9.8–33.1%) | 10.3% (3.9–21.2%) | 11.5% (2.4–30.2%) | 3% (0.1–15.8%) | 4.2% (1.6–9%) |
| 2001-02 | 405 | 40.3% (28.5–53%) | 21.1% (11.4–33.9%) | 8.8% (2.9–19.3%) | 4.2% (0.1–21.1%) | 9.7% (2–25.8%) | 6.6% (3.3–11.5%) |
| 2003-04 | 502 | 41.4% (29.8–53.8%) | 26.4% (15.3–40.3%) | 13.1% (5.8–24.2%) | 5.1% (0.6–17.3%) | 5.1% (0.6–17.3%) | 0.8% (0.1–3%) |
| 2005-06 | 421 | 18.9% (8–35.2%) | 37.2% (23–53.3%) | 16% (7.2–29.1%) | 3.8% (0.1–19.6%) | 8.8% (1.9–23.7%) | 2.2% (0.7–5%) |
| 2007-08 | 705 | 40.8% (29.6–52.7%) | 18.3% (10.1–29.3%) | 9.4% (3.1–20.7%) | 2.1% (0.1–11.1%) | 1.5% (0–8.3%) | 4.1% (2.4–6.6%) |
| 2009-10 | 857 | 31.9% (21.4–44%) | 19.8% (12.2–29.4%) | 19% (10.2–30.9%) | 1.9% (0–10.3%) | 1.3% (0–7.1%) | 0.8% (0.2–2%) |
| 2011-12 | 833 | 24.2% (11.1–42.3%) | 15% (9.1–22.7%) | 11.9% (5.9–20.8%) | 4.3% (0.5–14.8%) | 2.4% (0.3–8.3%) | 1.1% (0.3–2.5%) |
| 2013-14 | 834 | 14.3% (6.7–25.4%) | 15.4% (8.7–24.5%) | 11.2% (5.7–19.2%) | 3.9% (0.5–13.5%) | 2.4% (0.3–8.4%) | 1.1% (0.4–2.6%) |
| 2015-16 | 732 | 31% (17.6–47.1%) | 9.1% (3.4–18.7%) | 18.6% (9.7–30.9%) | 3.3% (0.1–17.2%) | 5.1% (1.4–12.5%) | 3.5% (2–5.6%) |
| 2017-18 | 640 | 29.3% (16.1–45.5%) | 18.5% (9.9–30%) | 9.1% (3.4–18.7%) | 0% (0–11.9%) | 1.7% (0–9.1%) | 0.5% (0.1–1.9%) |
| 2019-20 | 535 | 24.4% (12.4–40.3%) | 14.3% (6.4–26.2%) | 8.8% (3.3–18.2%) | 3.2% (0.1–16.7%) | 6.4% (1.3–17.5%) | 1% (0.2–3%) |

**Online supplemental table S6:** Patch test results with the waving/relaxing agents included in the “Hairdresser Series”, observed in female hairdressers (n=2678) and consumers (n=6244), resp., patch tested 1995–2020 in the departments of the IVDK. %pos(std.), age-stratified prevalence with accompanying 95% exact confidence interval (CI). **(A)** ammonium thioglycolate (ATG) 1% aqu.; **(B)** glyceryl thioglycolate (GMTG) 1% pet.

**(A) ATG**

| Period | n(test) | $\leq$ 20 y | 21–32 y | $>$ 32 y | $\leq$ 20 y | 21–32 y | $>$ 32 y |
| --- | --- | --- | --- | --- | --- | --- | --- |
|  |  | Hairdressers |  |  | Consumers |  |  |
| 2003-04 | 84 | 0% (0–21.8%) | 0% (0–36.9%) | 0% (0–45.9%) | 0% (0–36.9%) | 0% (0–45.9%) | 0% (0–8.6%) |
| 2005-06 | 403 | 0% (0–10.3%) | 0% (0–8.4%) | 2.1% (0.1–11.3%) | 0% (0–13.7%) | 0% (0–10.9%) | 1.8% (0.5–4.5%) |
| 2007-08 | 694 | 0% (0–4.8%) | 2.9% (0.4–10.2%) | 0% (0–6.7%) | 0% (0–7.5%) | 0% (0–5.5%) | 0.8% (0.2–2.3%) |
| 2009-10 | 854 | 0% (0–4.9%) | 0% (0–3.8%) | 4.8% (1–13.3%) | 2% (0.1–10.6%) | 0% (0–4.9%) | 0.2% (0–1.1%) |
| 2011-12 | 835 | 3% (0.1–15.8%) | 0% (0–3%) | 2.4% (0.3–8.2%) | 0% (0–7.7%) | 0% (0–4.2%) | 0.2% (0–1.2%) |
| 2013-14 | 841 | 0% (0–5.7%) | 4.3% (1.2–10.6%) | 2% (0.2–7%) | 2% (0–10.4%) | 1.2% (0–6.5%) | 0.2% (0–1.2%) |
| 2015-16 | 732 | 0% (0–8.2%) | 3% (0.4–10.4%) | 3.4% (0.4–11.7%) | 0% (0–11.9%) | 0% (0–4.5%) | 0.4% (0.1–1.6%) |
| 2017-18 | 637 | 0% (0–8.6%) | 1.6% (0–8.4%) | 0% (0–5.2%) | 3.4% (0.1–17.8%) | 1.7% (0–9.1%) | 0.5% (0.1–1.9%) |
| 2019-20 | 526 | 4.9% (0.6–16.5%) | 0% (0–6.7%) | 1.6% (0–8.4%) | 3.3% (0.1–17.2%) | 4.3% (0.5–14.8%) | 1% (0.2–3%) |

**(B) GMTG**

| Period | n(test) | $\leq$ 20 y | 21–32 y | $>$ 32 y | $\leq$ 20 y | 21–32 y | $>$ 32 y |
| --- | --- | --- | --- | --- | --- | --- | --- |
|  |  | Hairdressers |  |  | Consumers |  |  |
| 1995-96 | 366 | 41.9% (30.5–53.9%) | 30.2% (19.2–43%) | 20% (9.1–35.6%) | 0% (0–12.3%) | 6.2% (0.8–20.8%) | 0.8% (0–4.5%) |
| 1997-98 | 454 | 13.8% (6.5–24.7%) | 12.2% (4.6–24.8%) | 15.6% (6.5–29.5%) | 0% (0–11.9%) | 3.6% (0.4–12.5%) | 2.4% (0.8–5.6%) |
| 1999-2000 | 372 | 11.9% (4.9–22.9%) | 9.8% (3.3–21.4%) | 5.1% (1.1–14.1%) | 3.8% (0.1–19.6%) | 6.1% (0.7–20.2%) | 0.7% (0–3.9%) |
| 2001-02 | 406 | 7.4% (2.4–16.3%) | 5.4% (1.1–14.9%) | 5.1% (1.1–14.1%) | 0% (0–14.2%) | 3.2% (0.1–16.7%) | 0.6% (0–3.3%) |
| 2003-04 | 500 | 4.3% (0.9–12%) | 9.4% (3.1–20.7%) | 10% (3.8–20.5%) | 2.6% (0.1–13.5%) | 2.6% (0.1–13.8%) | 1.2% (0.3–3.6%) |
| 2005-06 | 417 | 0% (0–9.7%) | 7.3% (1.5–19.9%) | 8% (2.2–19.2%) | 0% (0–13.7%) | 2.9% (0.1–15.3%) | 0.4% (0–2.4%) |
| 2007-08 | 708 | 3.9% (0.8–11.1%) | 2.7% (0.3–9.5%) | 14.8% (6.6–27.1%) | 0% (0–7.3%) | 1.6% (0–8.4%) | 2% (0.9–4%) |
| 2009-10 | 857 | 4.1% (0.9–11.5%) | 2.2% (0.3–7.6%) | 9.7% (3.6–19.9%) | 0% (0–6.8%) | 0% (0–4.7%) | 0.8% (0.2–2%) |
| 2011-12 | 832 | 0% (0–10.6%) | 0.8% (0–4.5%) | 4.9% (1.3–12%) | 2.2% (0.1–11.5%) | 0% (0–4.3%) | 1.5% (0.6–3.1%) |
| 2013-14 | 835 | 1.6% (0–8.5%) | 1.1% (0–6%) | 8.1% (3.6–15.3%) | 0% (0–7%) | 1.2% (0–6.6%) | 1.3% (0.5–2.9%) |
| 2015-16 | 736 | 2.3% (0.1–12.3%) | 0% (0–5.4%) | 6.8% (1.9–16.5%) | 0% (0–11.6%) | 0% (0–4.5%) | 2% (0.9–3.7%) |
| 2017-18 | 635 | 0% (0–8.6%) | 0% (0–5.8%) | 9.1% (3.4–18.7%) | 6.9% (0.8–22.8%) | 0% (0–6.1%) | 1.6% (0.6–3.4%) |
| 2019-20 | 501 | 0% (0–9.3%) | 0% (0–6.7%) | 1.7% (0–8.9%) | 0% (0–11.6%) | 2.2% (0.1–11.8%) | 0.7% (0.1–2.6%) |

**Online supplemental table S7:** Patch test results with two preservatives included in the “Baseline Series”, observed in female hairdressers (n=2678) and consumers (n=6244), resp., patch tested 1995–2020 in the departments of the IVDK. %pos(std.), age-stratified prevalence with accompanying 95% exact confidence interval (CI). **(A)** Methylchloroisothiazolinone/methylisothiazolinone (MCI/MI) 0.01% aqu.; **(B)** methyldibromo glutaronitrile (MDBGN) 0.2% pet. (1997 to 2015) and 0.3% pet. (since 2016), respectively

**(A) MCI/MI**

| Period | n(test) | $\leq$ 20 y | 21–32 y | $>$ 32 y | $\leq$ 20 y | 21–32 y | $>$ 32 y |
| --- | --- | --- | --- | --- | --- | --- | --- |
|  |  | Hairdressers |  |  | Consumers |  |  |
| 1995-96 | 414 | 2.7% (0.3–9.3%) | 0% (0–5.7%) | 5.4% (0.7–18.2%) | 0% (0–10.9%) | 2.2% (0.1–11.8%) | 2.6% (0.7–6.5%) |
| 1997-98 | 493 | 0% (0–6%) | 2.3% (0.1–12%) | 11.4% (3.8–24.6%) | 0% (0–10.6%) | 1.6% (0–8.5%) | 2.5% (0.9–5.3%) |
| 1999-2000 | 399 | 3.4% (0.4–11.7%) | 0% (0–7%) | 1.8% (0–9.6%) | 0% (0–13.2%) | 0% (0–9.5%) | 3% (1–6.8%) |
| 2001-02 | 430 | 3.1% (0.4–10.8%) | 0% (0–6.7%) | 8.3% (2.8–18.4%) | 0% (0–11.9%) | 2.9% (0.1–15.3%) | 0% (0–2%) |
| 2003-04 | 524 | 4.5% (0.9–12.7%) | 0% (0–6.8%) | 4.8% (1–13.5%) | 2.7% (0.1–14.2%) | 2.1% (0.1–11.3%) | 0.4% (0–2.1%) |
| 2005-06 | 482 | 8.6% (1.8–23.1%) | 5.1% (0.6–17.3%) | 5.9% (1.2–16.2%) | 7.4% (0.9–24.3%) | 2.4% (0.1–12.9%) | 2.1% (0.8–4.5%) |
| 2007-08 | 797 | 5.4% (1.5–13.3%) | 2.7% (0.3–9.4%) | 1.6% (0–8.8%) | 0% (0–6.6%) | 1.3% (0–7%) | 1.8% (0.8–3.4%) |
| 2009-10 | 909 | 1.4% (0–7.6%) | 3.3% (0.7–9.2%) | 7.9% (2.6–17.6%) | 0% (0–7%) | 1.1% (0–6.2%) | 1.3% (0.5–2.6%) |
| 2011-12 | 913 | 14.3% (4.8–30.3%) | 5.1% (1.9–10.8%) | 11% (5.4–19.3%) | 2% (0.1–10.9%) | 4.2% (1.2–10.4%) | 4.4% (2.8–6.5%) |
| 2013-14 | 880 | 6.7% (1.8–16.2%) | 7.8% (3.2–15.4%) | 18.6% (11.6–27.6%) | 3.8% (0.5–13.2%) | 1.2% (0–6.4%) | 4.5% (2.8–6.7%) |
| 2015-16 | 752 | 2.5% (0.1–13.2%) | 9.4% (3.5–19.3%) | 14% (6.3–25.8%) | 6.9% (0.8–22.8%) | 2.3% (0.3–8.1%) | 2.7% (1.5–4.6%) |
| 2017-18 | 645 | 0% (0–9%) | 5.4% (1.1–14.9%) | 9.2% (3.5–19%) | 0% (0–11.9%) | 0% (0–6.2%) | 2.8% (1.4–4.9%) |
| 2019-20 | 517 | 0% (0–9%) | 0% (0–6.6%) | 3% (0.4–10.4%) | 0% (0–11.6%) | 0% (0–7.5%) | 1.8% (0.6–4.1%) |

**(B) MDBGN**

| Period | n(test) | $\leq$ 20 y | 21–32 y | $>$ 32 y | $\leq$ 20 y | 21–32 y | $>$ 32 y |
| --- | --- | --- | --- | --- | --- | --- | --- |
|  |  | Hairdressers |  |  | Consumers |  |  |
| 1997-98^a)^ | 370 | 6.7% (1.4–18.3%) | 3.2% (0.1–16.7%) | 13.3% (3.8–30.7%) | 8.3% (1–27%) | 0% (0–8.2%) | 4.2% (1.8–8%) |
| 1999-2000^a)b)^ | 155 | 5.3% (0.1–26%) | 3.8% (0.1–19.6%) | 21.1% (6.1–45.6%) | 0% (0–30.8%) | 0% (0–19.5%) | 1.6% (0–8.7%) |
| 2001-02^b)^ | 39 | 0% (0–60.2%) | 0% (0–70.8%) | 0% (0–70.8%) | 0% (0–70.8%) | 0% (0–70.8%) | 4.3% (0.1–21.9%) |
| 2003-04^b)^ | 12 | 0% (0–97.5%) | 0% (0–70.8%) | 33.3% (0.8–90.6%) | 0% (0–97.5%) |  | 0% (0–60.2%) |
| 2005-06^a)^ | 585 | 2.7% (0.1–14.2%) | 8.3% (2.3–20%) | 14% (6.3–25.8%) | 9.7% (2–25.8%) | 0% (0–6.8%) | 4.4% (2.6–7.1%) |
| 2007-08^a)c)^ | 798 | 1.4% (0–7.4%) | 8.9% (3.6–17.4%) | 4.8% (1–13.3%) | 0% (0–6.7%) | 0% (0–4.7%) | 2% (0.9–3.7%) |
| 2009-10^c)^ | 909 | 1.4% (0–7.5%) | 1.1% (0–6%) | 4.7% (1–13.1%) | 2% (0–10.4%) | 2.3% (0.3–8.1%) | 2.2% (1.1–3.8%) |
| 2011-12^c)^ | 911 | 0% (0–10.3%) | 0.9% (0–4.7%) | 4.4% (1.2–10.9%) | 0% (0–7.3%) | 1.1% (0–5.7%) | 1.5% (0.7–3%) |
| 2013-14^c)^ | 880 | 1.7% (0–8.9%) | 2.2% (0.3–7.7%) | 3.9% (1.1–9.6%) | 3.8% (0.5–13.2%) | 1.2% (0–6.4%) | 1.8% (0.8–3.5%) |
| 2015-16^c)a)^ | 755 | 0% (0–8.6%) | 1.5% (0–8.2%) | 5.1% (1.1–14.1%) | 3.4% (0.1–17.8%) | 1.1% (0–6.2%) | 2.5% (1.3–4.4%) |
| 2017-18^a)^ | 650 | 0% (0–8.8%) | 3.5% (0.4–12.1%) | 2.9% (0.4–10.2%) | 0% (0–12.3%) | 1.7% (0–9.1%) | 5.8% (3.7–8.6%) |

^a)^ MDBGN 0.3% pet.; ^b)^ MDBGN + 2-phenoxyethanol 1:4 1% pet.; ^c)^ MDBGN 0.2% pet.
