# Supplementary figures and images for: Contact Allergy to Ingredients of Hair Cosmetics Associated with Occupational and Non‐Occupational Exposure—Trends from 1995 to 2020 in Central Europe, with or without Regulation
Source: Contact Dermatitis. 2025 Dec 30;94(4):347–63. doi: 10.1111/cod.70079 (PMC12956424; doi:10.1111/cod.70079)

% of females tested

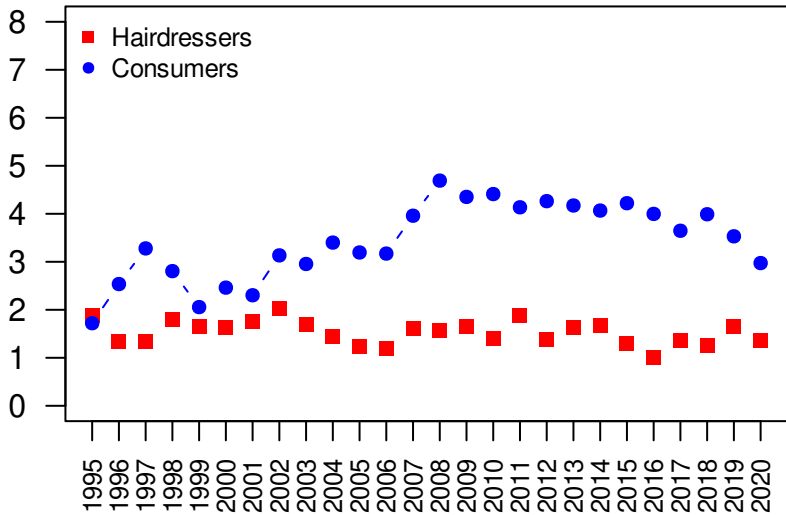

Supplement: Supplementary file 2 — Figure S1: The share of hairdressers and clients, respectively, among all patch tested female patients consulting the departments of the IVDK between 1995 and 2020. [file COD-94-347-s006.pdf]

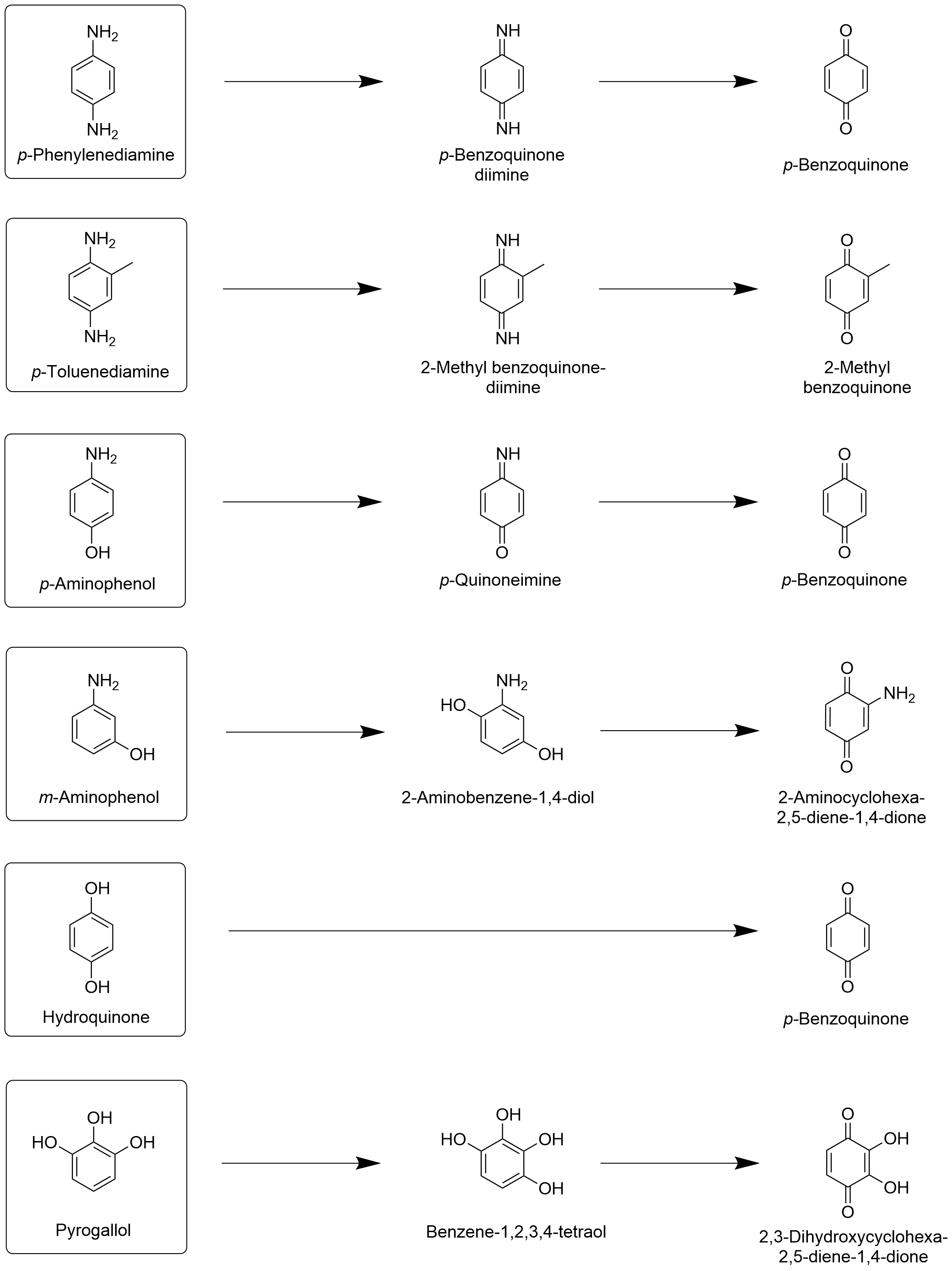

Supplement: Supplementary file 8 — Figure S7: Transformation of structurally related and unrelated chemicals: examples of possible formation of benzoquinone or benzoquinone derivatives. [file COD-94-347-s005.tif]
