# Supplementary material for: Contact Allergy to Ingredients of Hair Cosmetics Associated with Occupational and Non‐Occupational Exposure—Trends from 1995 to 2020 in Central Europe, with or without Regulation
Source: Contact Dermatitis. 2025 Dec 30;94(4):347–63. doi: 10.1111/cod.70079 (PMC12956424; doi:10.1111/cod.70079)
Supplement: Supplementary file 3 — Figure S2: Time trend of positive patch test reactions (crude, age‐stratified prevalences) to p‐phenylenediamine (PPD) 1% pet. in female hairdressers (left) and female consumers (right) consulting the departments of the IVDK between 1995 and 2020. As this allergen has not been regularly tested during 5 years, between 01/2005 and 12/2009, these years were excluded in the graph. [file COD-94-347-s003.pdf]

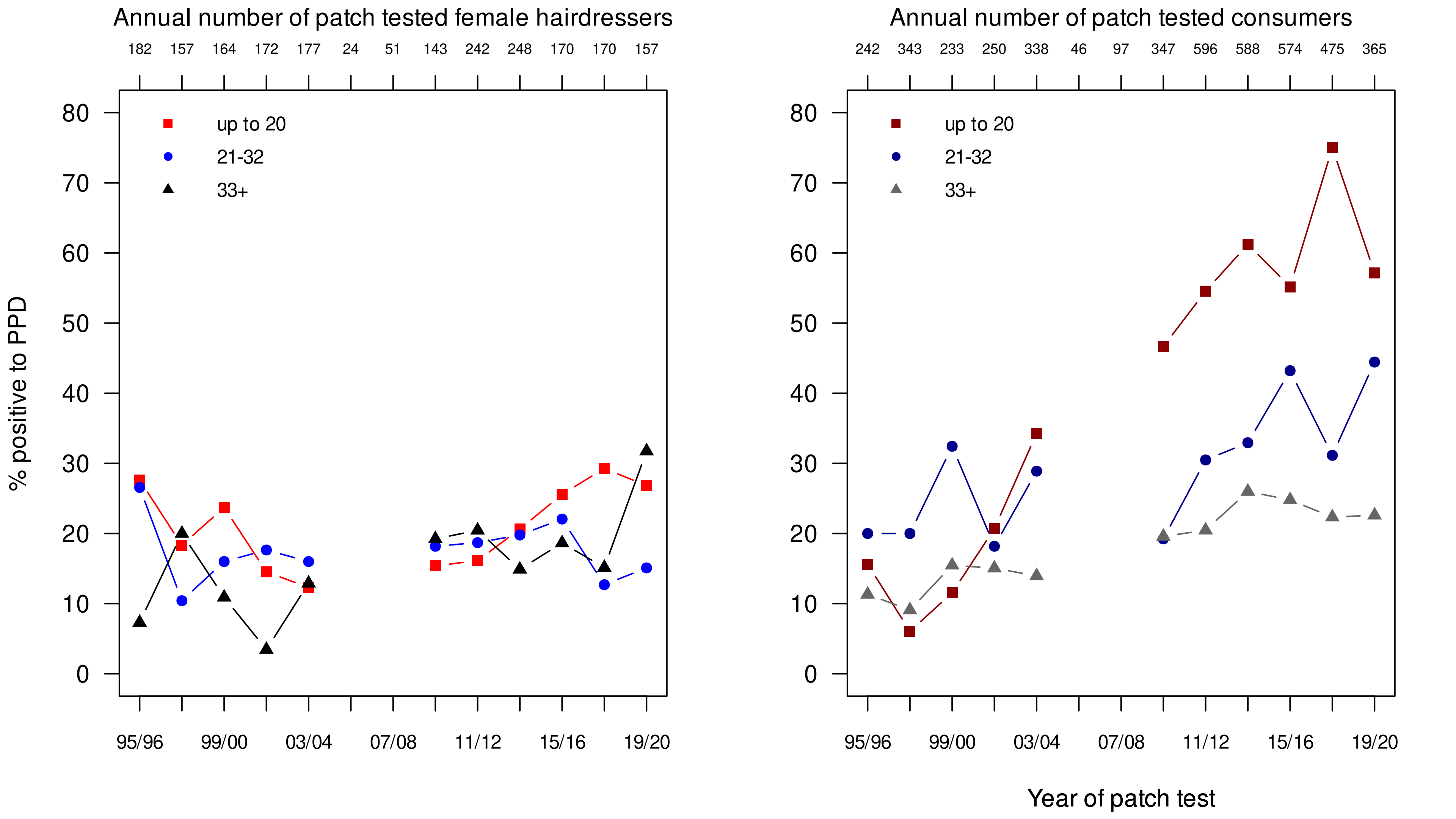

Annual number of patch tested female hairdressers
182 157 164 172 177 24 51 143 242 248 170 170 157
up to 20
21-32
33+
% positive to PPD
0 10 20 30 40 50 60 70 80
95/96 99/00 03/04 07/08 11/12 15/16 19/20
Annual number of patch tested consumers
242 343 233 250 338 46 97 347 596 588 574 475 365
up to 20
21-32
33+
0 10 20 30 40 50 60 70 80
95/96 99/00 03/04 07/08 11/12 15/16 19/20
Year of patch test
