# Supplementary material for: Contact Allergy to Ingredients of Hair Cosmetics Associated with Occupational and Non‐Occupational Exposure—Trends from 1995 to 2020 in Central Europe, with or without Regulation
Source: Contact Dermatitis. 2025 Dec 30;94(4):347–63. doi: 10.1111/cod.70079 (PMC12956424; doi:10.1111/cod.70079)
Supplement: Supplementary file 4 — Figure S3: Time trend of positive patch test reactions (crude, age‐stratified prevalences) to pyrogallol 1% pet. in female hairdressers (left) and female consumers (right) consulting the departments of the IVDK between 1995 and 2020. [file COD-94-347-s001.pdf]

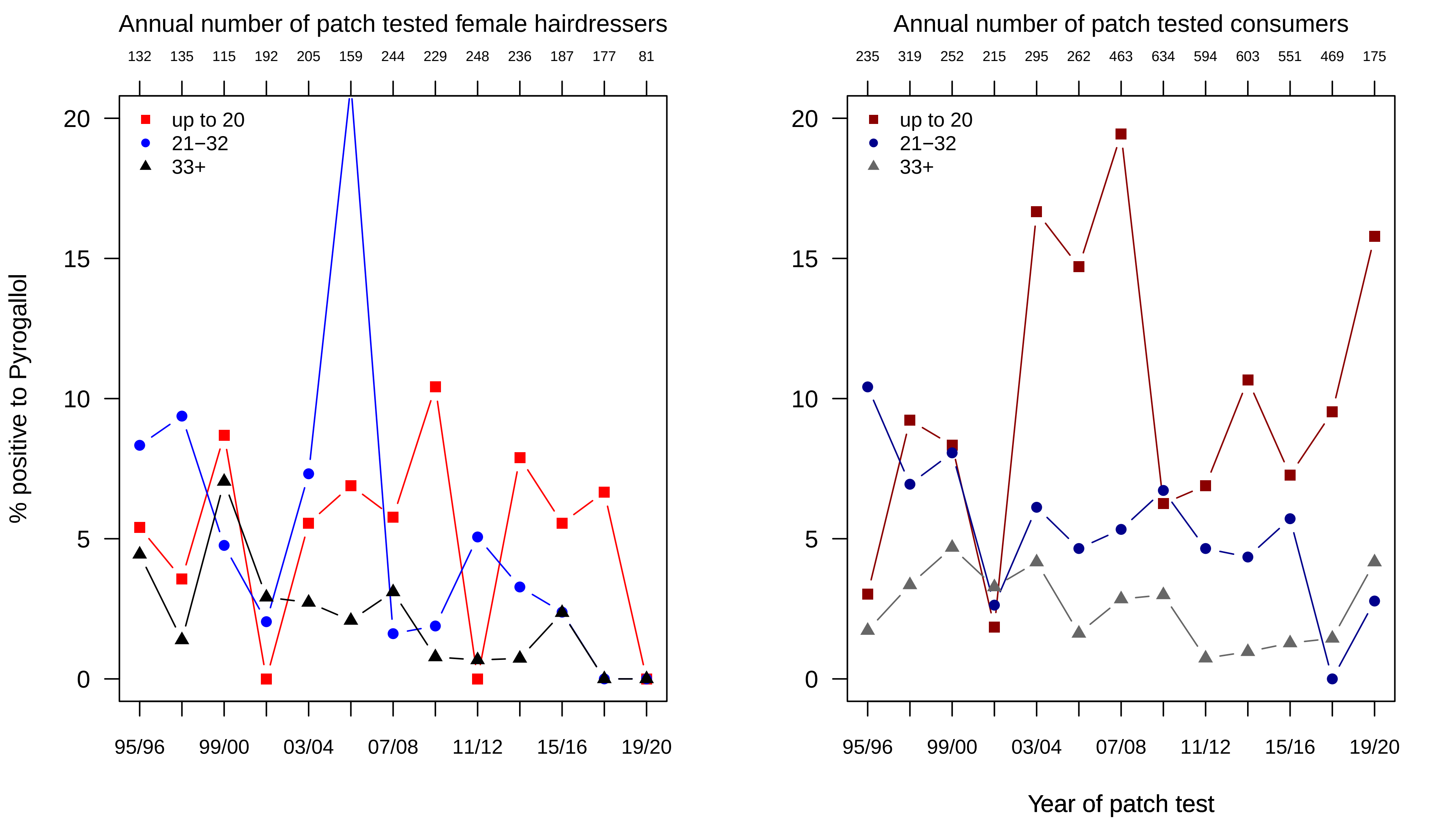
Annual number of patch tested female hairdressers
132 135 115 192 205 159 244 229 248 236 187 177 81
up to 20
21−32
33+
% positive to Pyrogallol
0
5
10
15
20
95/96 99/00 03/04 07/08 11/12 15/16 19/20
Annual number of patch tested consumers
235 319 252 215 295 262 463 634 594 603 551 469 175
up to 20
21−32
33+
Year of patch test
