# Supplementary material for: Contact Allergy to Ingredients of Hair Cosmetics Associated with Occupational and Non‐Occupational Exposure—Trends from 1995 to 2020 in Central Europe, with or without Regulation
Source: Contact Dermatitis. 2025 Dec 30;94(4):347–63. doi: 10.1111/cod.70079 (PMC12956424; doi:10.1111/cod.70079)
Supplement: Supplementary file 5 — Figure S4: Time trend of positive patch test reactions (crude, age‐stratified prevalences) to ammonium thioglycolate (ATG) 1% aqu. in female hairdressers (left) and female consumers (right) consulting the departments of the IVDK between 2004 and 2020. [file COD-94-347-s002.pdf]

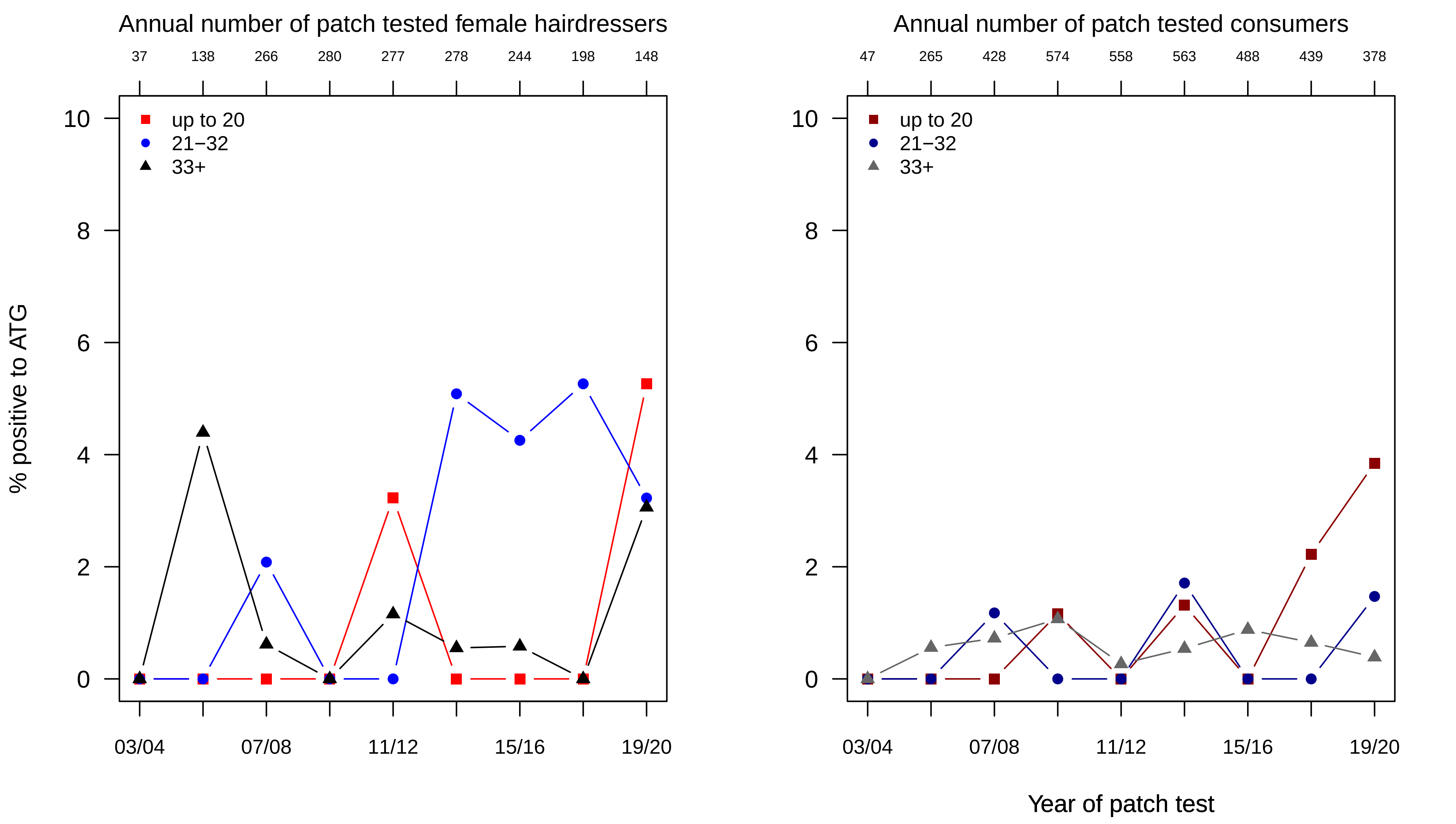

Annual number of patch tested female hairdressers
37 138 266 280 277 278 244 198 148
up to 20
21−32
33+
% positive to ATG
0 2 4 6 8 10
03/04 07/08 11/12 15/16 19/20
Annual number of patch tested consumers
47 265 428 574 558 563 488 439 378
up to 20
21−32
33+
0 2 4 6 8 10
03/04 07/08 11/12 15/16 19/20
Year of patch test
