# Supplementary material for: Contact Allergy to Ingredients of Hair Cosmetics Associated with Occupational and Non‐Occupational Exposure—Trends from 1995 to 2020 in Central Europe, with or without Regulation
Source: Contact Dermatitis. 2025 Dec 30;94(4):347–63. doi: 10.1111/cod.70079 (PMC12956424; doi:10.1111/cod.70079)
Supplement: Supplementary file 6 — Figure S5: Time trend of positive patch test reactions (crude, age‐stratified prevalences) to methylchloroisothiazolinone/methylisothiazolinone 3:1 (MCI/MI) 0.01% aqu. in female hairdressers (left) and female consumers (right) consulting the departments of the IVDK between 2004 and 2020. [file COD-94-347-s004.pdf]

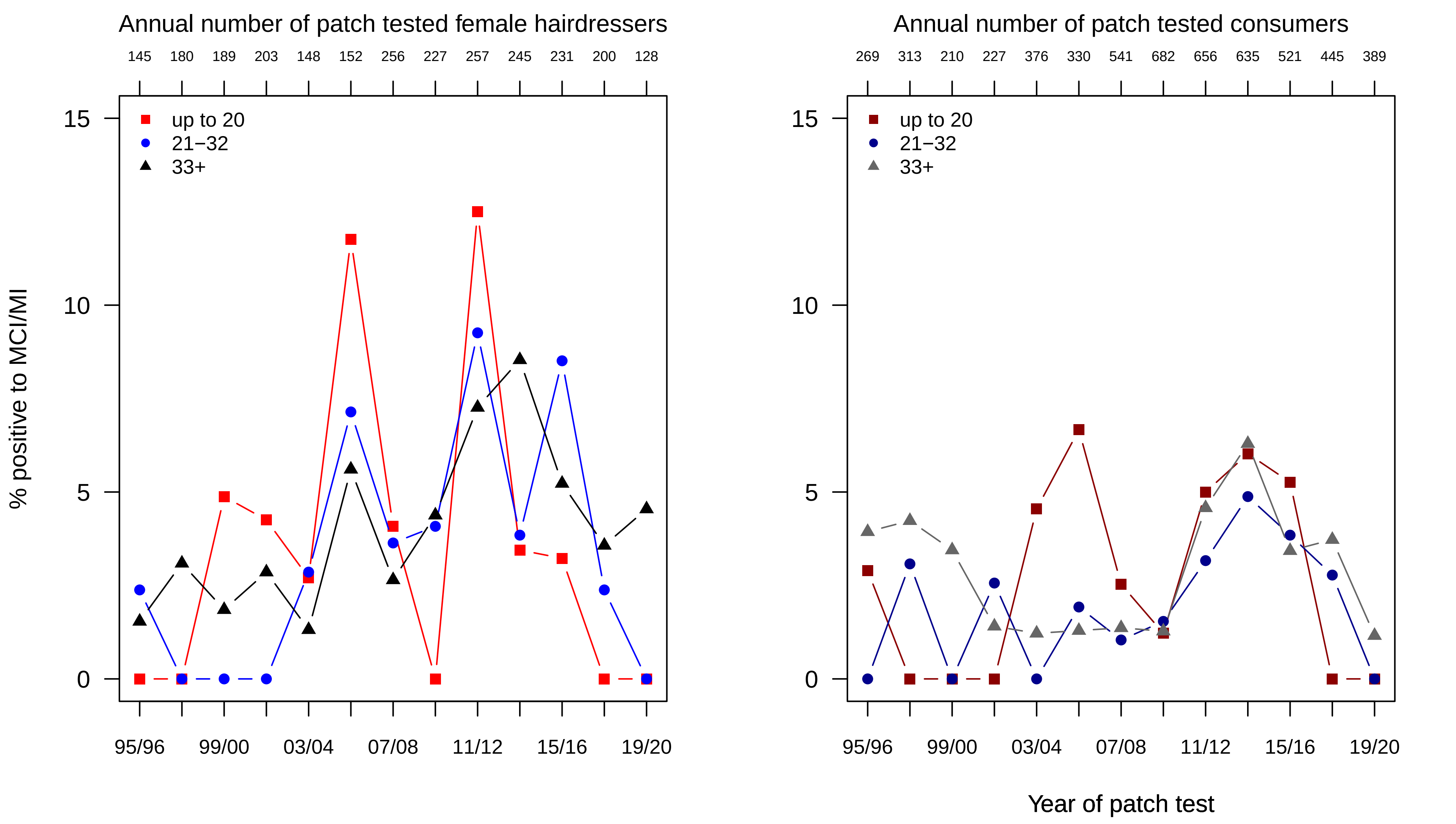

Annual number of patch tested female hairdressers
145 180 189 203 148 152 256 227 257 245 231 200 128
up to 20
21−32
33+
% positive to MCI/MI
15
10
5
0
95/96 99/00 03/04 07/08 11/12 15/16 19/20
Annual number of patch tested consumers
269 313 210 227 376 330 541 682 656 635 521 445 389
up to 20
21−32
33+
15
10
5
0
95/96 99/00 03/04 07/08 11/12 15/16 19/20
Year of patch test
