# Supplementary material for: Contact Allergy to Ingredients of Hair Cosmetics Associated with Occupational and Non‐Occupational Exposure—Trends from 1995 to 2020 in Central Europe, with or without Regulation
Source: Contact Dermatitis. 2025 Dec 30;94(4):347–63. doi: 10.1111/cod.70079 (PMC12956424; doi:10.1111/cod.70079)
Supplement: Supplementary file 7 — Figure S6: Time trend of positive patch test reactions (crude, age‐stratified prevalences) to methyldibromo glutaronitrile (MDBGN) 0.2 or 0.3% pet. (see text in “results”) in female hairdressers (left) and female consumers (right) consulting the departments of the IVDK between 2004 and 2020. [file COD-94-347-s007.pdf]

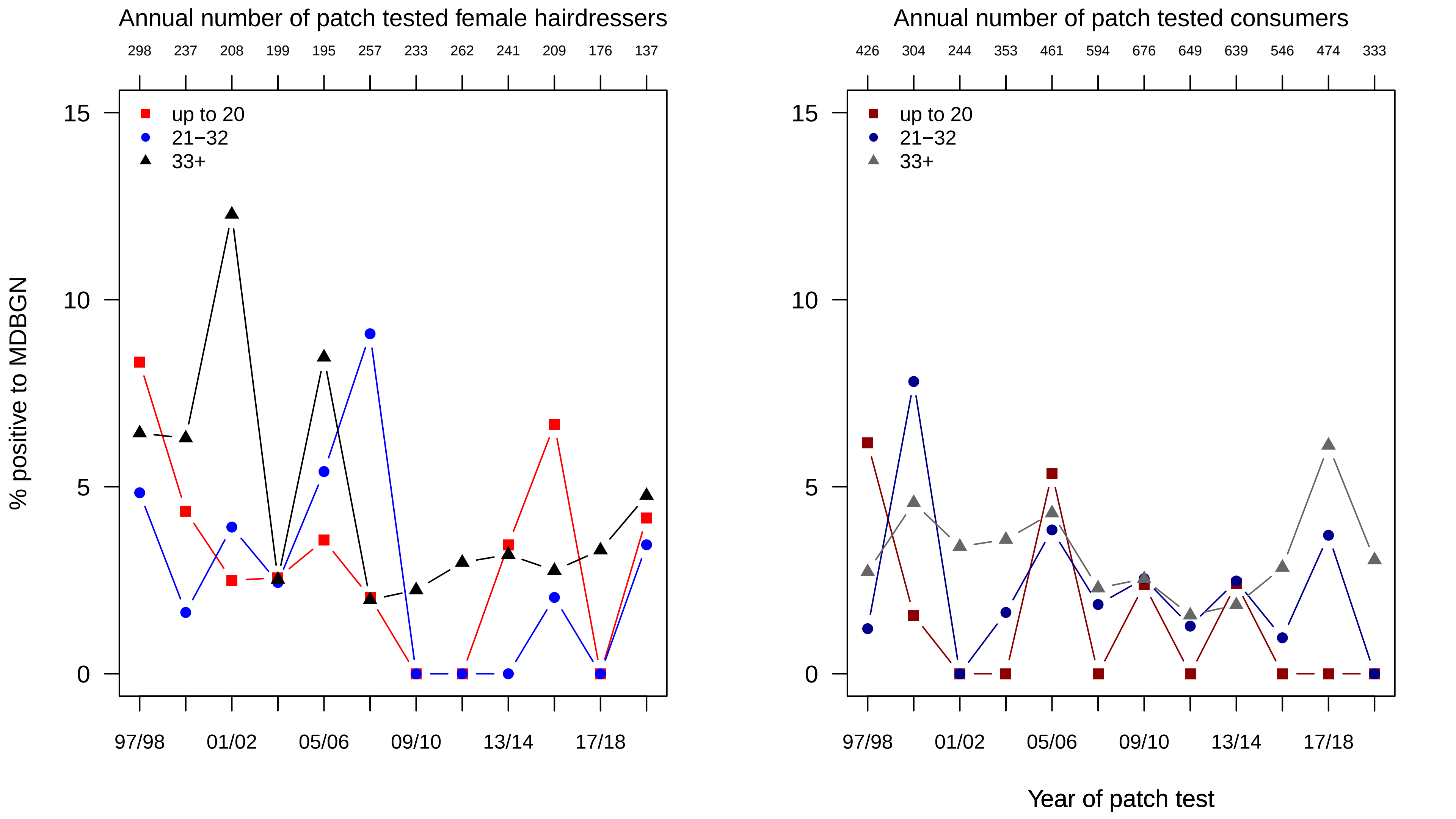
Annual number of patch tested female hairdressers
298 237 208 199 195 257 233 262 241 209 176 137
up to 20
21−32
33+
15
10
5
0
97/98 01/02 05/06 09/10 13/14 17/18
% positive to MDBGN
Annual number of patch tested consumers
426 304 244 353 461 594 676 649 639 546 474 333
up to 20
21−32
33+
15
10
5
0
97/98 01/02 05/06 09/10 13/14 17/18
Year of patch test
